# Bioinformatics integration reveals key genes associated with mitophagy in myocardial ischemia-reperfusion injury

**DOI:** 10.1186/s12872-024-03834-x

**Published:** 2024-03-27

**Authors:** Zhian Chen, Tianying Liu, Hao Yuan, Han Sun, Sitong Liu, Shuai Zhang, Li Liu, Shuang Jiang, Yong Tang, Zhi Liu

**Affiliations:** Department of Clinical Medicine, Changchun University of Chinese Medicine, No. 1035, Boshuo Road, Nanguan District, Changchun, 130,117 Jilin Province China

**Keywords:** Ischemia–reperfusion injury, Mitophagy, Bioinformatics, Hub genes

## Abstract

**Background:**

Myocardial ischemia is a prevalent cardiovascular disorder associated with significant morbidity and mortality. While prompt restoration of blood flow is essential for improving patient outcomes, the subsequent reperfusion process can result in myocardial ischemia–reperfusion injury (MIRI). Mitophagy, a specialized autophagic mechanism, has consistently been implicated in various cardiovascular disorders. However, the specific connection between ischemia–reperfusion and mitophagy remains elusive. This study aims to elucidate and validate central mitophagy-related genes associated with MIRI through comprehensive bioinformatics analysis.

**Methods:**

We acquired the microarray expression profile dataset (GSE108940) from the Gene Expression Omnibus (GEO) and identified differentially expressed genes (DEGs) using GEO2R. Subsequently, these DEGs were cross-referenced with the mitophagy database, and differential nucleotide sequence analysis was performed through enrichment analysis. Protein–protein interaction (PPI) network analysis was employed to identify hub genes, followed by clustering of these hub genes using cytoHubba and MCODE within Cytoscape software. Gene set enrichment analysis (GSEA) was conducted on central genes. Additionally, Western blotting, immunofluorescence, and quantitative polymerase chain reaction (qPCR) analyses were conducted to validate the expression patterns of pivotal genes in MIRI rat model and H9C2 cardiomyocytes.

**Results:**

A total of 2719 DEGs and 61 mitophagy-DEGs were identified, followed by enrichment analyses and the construction of a PPI network. HSP90AA1, RPS27A, EEF2, EIF4A1, EIF2S1, HIF-1α, and BNIP3 emerged as the seven hub genes identified by cytoHubba and MCODE of Cytoscape software. Functional clustering analysis of HIF-1α and BNIP3 yielded a score of 9.647, as determined by Cytoscape (MCODE). In our MIRI rat model, Western blot and immunofluorescence analyses confirmed a significant elevation in the expression of HIF-1α and BNIP3, accompanied by a notable increase in the ratio of LC3II to LC3I. Subsequently, qPCR confirmed a significant upregulation of HIF-1α, BNIP3, and LC3 mRNA in the MIRI group. Activation of the HIF-1α/BNIP3 pathway mediates the regulation of the degree of Mitophagy, thereby effectively reducing apoptosis in rat H9C2 cardiomyocytes.

**Conclusions:**

This study has identified seven central genes among mitophagy-related DEGs that may play a pivotal role in MIRI, suggesting a correlation between the HIF-1α/BNIP3 pathway of mitophagy and the pathogenesis of MIRI. The findings highlight the potential importance of mitophagy in MIRI and provide valuable insights into underlying mechanisms and potential therapeutic targets for further exploration in future studies.

**Supplementary Information:**

The online version contains supplementary material available at 10.1186/s12872-024-03834-x.

## Introduction

Myocardial ischemia–reperfusion injury (MIRI) is a prevalent occurrence in cardiac diseases, referring to the damage caused by inadequate blood supply during ischemia and further exacerbated upon reperfusion [1, 2]. IRI occurs in situations such as cardiac surgery, coronary artery disease, and heart transplantation, which is closely associated with pathophysiological processes, including myocardial cell injury, inflammatory response and oxidative stress [3]. Studies have found that myocardium undergoes various types of damage during ischemia–reperfusion, including cell membrane rupture, mitochondrial dysfunction [4], oxidative stress and activation of inflammatory response, leading to cardiomyocyte death, tissue necrosis, inflammation and myocardial dysfunction [5, 6].

Numerous studies have been dedicated to unraveling the mechanisms of IRI to identify new therapeutic strategies [7]. Among these strategies, targeting mitophagy as a cellular self-repair mechanism has received significant attention. Mitophagy is a lysosome-mediated autophagic process that involves engulfing damaged or aged mitochondria for degradation, thereby maintaining a healthy mitochondrial status and cellular bioenergetics [8]. In neurodegenerative diseases, such as Alzheimer’s disease, improper regulation of mitophagy can lead to the clustering of many damaged mitochondria and a subsequent decrease in mitochondrial function [9, 10]. These damaged mitochondria release higher levels of free radicals and cytotoxic substances, causing neuronal cell damage and apoptosis, which accelerates the progression of the disease [11]. Mitophagy plays a multifaceted role in tumorigenesis and therapy. In some cases, it promotes tumor cell survival and growth, thereby facilitating tumor progression [12, 13]. However, other studies have demonstrated that mitophagy in tumor cells may have cytotoxic effects, offering a potential novel strategy for antitumor therapy [14]. Moreover, mitophagy also significantly influences metabolic diseases. In the context of stroke, mitophagy has been found to play a pivotal role in protecting brain cells from ischemia–reperfusion injury [15, 16]. Moderately induced mitophagy effectively removes damaged mitochondria, reduces intracellular oxidative stress, and mitigates cell death, ultimately helping to limit stroke-induced brain tissue damage [17]. Overall, understanding the complex role of mitophagy in various diseases can pave the way for innovative therapeutic approaches and shed light on potential treatment strategies.

Currently, research on mitophagy in myocardial ischemia–reperfusion is relatively limited. To fill this gap, we employed bioinformatics and experimental research to identify DEGs in MIRI rats. To our knowledge, this is the first study to utilize bioinformatics and machine learning algorithms to research the effects of mitophagy on MIRI. It provides insights for new therapeutic strategies and lays a solid theoretical groundwork for forthcoming pioneering investigations.

## Materials and methods

### Data source

**Fig. 1:**
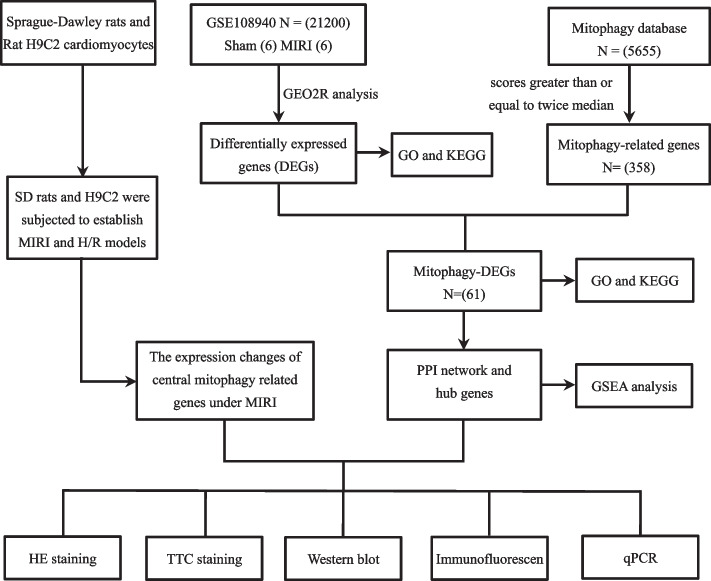
The analysis workflow flowchart

GSE108940 was obtained from the Gene Expression Omnibus (GEO) database (http://www.ncbi.nlm.nih.gov/geo). GSE108940 consists of 24 groups, from which 6 sham groups and 6 I/R groups were selected for analysis. The sham groups consisted of rats that underwent sham surgery, while the I/R groups consisted of rats with myocardial ischemia–reperfusion injury models. Mitophagy-related genes were obtained from Genecards (https://www.genecards.org/Search/Keyword?queryString=mitophagy), which included 5642 genes associated with mitosis. However, we only selected 358 relevant genes using a filtering mechanism with a correlation score ≥ 2 times the median. Principal Component Analysis (PCA) enables new variables to capture as many features of the original variables as possible while reducing dimensionality. Using the GEO2R analysis tool, we obtained DEGs with a statistical cutoff criteria of |logFC|> 0.58 and *P.adj* < 0.05. We then employed the ggplot function in R software to create heatmaps and volcano plots that depict data. The data processing workflow is illustrated in Fig. 1.

### Identification of DEGs and mitophagy-DEGs

GEO2R (www.ncbi.nlm.nih.gov/geo/ge2r) is a online analysis tool that utilizes the GEO query and Limma packages. We used GEO2R with a *P.adj* < 0.05 and |logFC|> 0.58 to identify DEGs. We then constructed VEEN diagrams to visualize the DEGs related to mitophagy among the identified 2719 DEGs and 358 mitophagy-related genes. The Venn diagram was created using the online tool available at the Venn website (http://bioinformatics.psb.ugent.be/Webtools/Venn/).

### Functional enrichment analysis

We employed GO (Gene Ontology) and the KEGG (Kyoto Encyclopedia of Genes and Genomes) for gene regulation and function analysis. This analysis was conducted by the clusterProfiler package (version 4.4.4) with a significance cutoff of *P.adj* < 0.05. Finally, we visualized the functional enrichment analysis results with the GO plot and ggplot packages in R software (version 4.2.1).

### GSEA enrichment analysis

We conducted hub gene enrichment analysis using GSEA (version 4.1.0), which distinguishes itself from traditional methods by enabling gene expression assessment in different subgroups and providing associated enrichment messages. For the analysis, we selected the c2.all.v2022.1.Hs.symbols.gmt [Curated/Pathway] (6449) database and performed 1000 permutations using a phenotype permutation method, while keeping all other settings at their default values. Gene pathways exhibiting |NES|> 1, *p* < 0.05, and FDR < 0.01 were deemed statistically significant.

### PPI network and hub gene analysis

The STRING is a dependable database utilized to explore protein relationships, encompassing direct binding interactions and regulatory pathways. For protein interaction analysis and identification of crucial protein genes associated with mitophagy-DEGs, we built a PPI network using STRING, considering interactions with a score > 0.4. PPI network was generated through Cytoscape version 3.8.2 [18], and significant modules were identified using MCODE, a Cytoscape plugin that applies specific criteria including degree cut-off = 2, MCODE scores > 5, max depth = 100, k-score = 2, and node score cutoff = 0.2. Lastly, we performed cytoHubba analysis to identify hub genes by applying the MCC method to rank the top six genes in the network.

### Animals and H9C2 cardiomyocytes model

SPF-rated male SD rats (8 weeks old, weighing 220 ± 20 g) were purchased from Changchun Yisi Experimental Animal Technology Co Ltd (Jilin, China) under license No. SCXK (Ji) -2020–0002. Rat H9C2 myocardial cells (batch number: STCC30008G) were procured from Servicebio Technology Co., Ltd., located in Wuhan, China. All rats were housed in the SPF-rated rat rearing room at the Animal Centre of Changchun University of Traditional Chinese Medicine and were kept under standard conditions: 25 °C, 12 h dark/light cycle. Animal studies were conducted following the Guide for the Care and Use of Laboratory Animals. They were approved by the Animal Experimentation Ethics Committee of Changchun University of Traditional Chinese Medicine (2022526).

Rats were divided into Sham and MIRI groups according to random number table method after 7 days of acclimatization feeding. Twenty-four hours after the last feeding, rats were anaesthetised by inhalation with 3% isoflurane (1 L/minute) prior to surgery, with 1% isoflurane as a maintenance dose [19]. The surgical site was meticulously disinfected using a solution of 70% ethanol combined with povidone-iodine, the rats' II-lead ECG was collected with an ECG machine, and the trachea was cut and connected to a ventilator [20]. The trachea was cut and connected to the ventilator. The skin was incised longitudinally at the left edge of the sternum, the tissues were sequentially separated to expose the heart, and ligation of the left coronary artery at the midsection of the left anterior descending (LAD) branch. The success of the ligation was determined by an elevated T-wave or ST-segment elevation in lead II, a darkening of the heart surface below the ligature line and a weakened pulsation. Ligation for 30 min, followed by perfusion for 120 min. In Sham group, the same anesthetic opening method was used as above, but only the wire was threaded, not ligated.

Rat H9C2 cardiomyocytes in the logarithmic growth phase were randomly divided into control (Con) group, hypoxia/reoxygenation (H/R) group, and YC-1 (HIF-1α inhibitor,S7958, Selleck, China) group. The Con group served as the untreated control. In the H/R group, cells were subjected to 4 h of hypoxia followed by 2 h of reoxygenation in tri-gas incubator (E5018,Beyotime, China) to establish the hypoxia/reoxygenation model [21]. The YC-1 group involved establishing the H/R model under the condition of HIF-1α inhibition.

### Tissue collecting and processing

After completing the perfusion in the MIRI group, changes in their electrocardiogram were observed, followed by the collection of myocardial tissue from both the MIRI and sham group rats. Twelve samples from MIRI and Sham rats were used for TTC staining, HE staining, western blot, immunofluorescence and qPCR analysis.

### Assessment of myocardial tissue by HE and TTC

HE staining was employed to assess the myocardial tissue following standard protocols. The infarcted region of the heart tissue was excised.After paraffin embedding, the tissue was serially sectioned into 0.5 μm-thick slices. Subsequently dewaxed, hydrated, stained and sealed. Images were captured using light microscope at 40 × 10 magnification, and the results were analyzed.

TTC staining was employed to assess myocardial infarction severity. After obtaining rat heart samples, the hearts were washed with 4 °C PBS and placed in a -80 °C freezer for 15 min. Subsequently, the frozen hearts were removed, myocardial tissues were placed in heart molds, and the thickness was adjusted to 2 mm for slicing. These slices were then immersed in a 2% TTC solution and incubated at 37 °C in a light-protected incubator for 15 to 30 min for staining. After staining, the samples were fixed with a 10% formaldehyde solution. Photographs were taken after 24 h and the infarct area was calculated.

### Western blotting

Western Blot detected expression of HIF-1α, BNIP3, LC3-II, and LC3-I. Each group took 0.3 g of cardiac tissue, added 1 ml of RIPA lysis buffer (P0013B, Beyotime, China) and homogenized using an electric homogenizer. The mixture was centrifuged at 12000 r/min for 12 min at 4℃, and supernatant was carefully retrieved. Protein concentration was determined using the BCA protein quantification method. Protein concentration was adjusted using the loading buffer (P0015, Beyotime, China) and lysis buffer, and 10 μg of protein was loaded. Electrophoresis was conducted consistently at 80 V for a duration of 2.5 h, and electrophoresis was stopped when Bromophenol blue reached the bottom of the gel [22]. Transferring was done by cutting the target bands according to the protein marker. Blocking was performed by shaking with blocking solution (P0216-300 g, Beyotime, China) for 1 h. After blocking, the membrane was washed 5 times with TBST (T1086, Solarbio, China) for 3 min each. The PVDF membrane (IPVH00010, Merck, Germany) was incubated with the primary antibody (anti-HIF-1α, GB111339, anti-BNIP3, GB111204, anti-LC3 A/B, GB11124, Servicebio, China) solution at 4 °C overnight, followed by immersion in the secondary antibody (anti-rabbit,bs-80295G-HRP,Bioss,China) solution and shaking for 60 min. Imaging in gel imaging system, and Image J was employed for the analysis.

Upon enzymatic dissociation with trypsin (G4012,servicebio,China), adherent cardiomyocytes were suspended, harvested, and subsequently counted. Cells were then lysed using the appropriate volume of RIPA lysis buffer to facilitate protein release. The ensuing experimental procedures, including the reagents utilized, were in alignment with those established for myocardial tissue Western blot analysis. Chemiluminescent substrates were employed for signal development, captured either by an imaging system or film in a darkroom setting. The band intensity on the membrane was analyzed to qualitatively or quantitatively assess the target protein.

### Immunofluorescence

Transection of the whole heart, with the left and right ventricles used for fixation in the transverse section of the heart, has a direct correlation with the partial area at risk. Heart tissue was fixed overnight in 4% paraformaldehyde. The tissues underwent dehydration, were subsequently embedded in paraffin, and then sectioned into slices measuring 5 mm in thickness. The sections were then baked and subjected to deparaffinization using xylene, absolute ethanol, and a gradient of alcohol in sequential order. Following deparaffinization, antigen retrieval was carried out at 97℃ for 30 min using antigen retrieval solution (P0088, Beyotime, China). Before blocking, tissue autofluorescence quenching reagent (G1221, Servicebio, China) was applied, followed by blocking with 3% BSA (4240GR100, BioFroxx, China) for 30 min. After incubating with the primary antibody, the fluorescent secondary antibody (anti-rabbit antibody, GB22303, Servicebio, China) was added and incubated for 50 min in dark. Staining results were analyzed using Image J after being photographed with an orthogonal fluorescence microscope.

### Quantitative real-time polymerase chain reaction

Total RNA was collected from rat myocardial tissue using the SPARK easy Improved Tissue RNA Kit (Spark Jade, AC0202, China). RNA purity was assessed using the NanoPhotometer N120. Subsequently, total RNA was subjected to reverse transcription using the SPARKscript II RT Plus Kit (Spark Jade, AG0304, China). Finally, the reaction was performed on a real-time PCR instrument (ABI, 7300 plus, America) [20]. LC3 protein expression level and localization status are often used as indicators to evaluate cellular autophagic activity. By detecting changes in the expression levels and ratios of LC3-I and LC3-II, the dynamic changes in autophagy initiation, progression, and termination can be determined. Therefore, we also included LC3 in our qPCR experiments. GAPDH was used as a positive control to quantify the expression levels of various samples. Details regarding the primers and probes are provided in Table 1.

**Table 1:** The primers used for qPCR

Gene	Forward Primer sequence (5' → 3')	Forward Primer sequence (5' → 3')
HIF-1α	ACCTTCATCGGAAACTCCAAAG	ACTGTTAGGCTCAGGTGAACT
BNIP3	TCCTGGGTAGAACTGCACTTC	GCTGGGCATCCAACAGTATTT
LC3	TCCGAGAAGACCTTCAAACAGC	AAGAAGGCTTGGTTAGCATTGAG
GAPDH	CTGGAGAAACCTGCCAAGTATG	GGTGGAAGAATGGGAGTTGCT

### Flow cytometric analysis of cardiomyocyte apoptosis

Following cell culture supernatant collection, trypsin without EDTA (G4011, Servicebio, China) was applied to digest the adherent cells, which were then pooled with the aforementioned supernatant. This mixture was subjected to centrifugation at 500 g, 4℃ for 5 min to pellet the cells. The resultant cell pellet was washed twice with pre-cooled PBS (G4202, Servicebio, China), each time followed by centrifugation at 500 g, 4℃ for 5 min. Cells were then gently resuspended in pre-cooled 1 × Binding Buffer (G1512-3, Servicebio, China) to adjust the cell concentration to 1–5 × 10^6/mL. To this, 100 µL of cell suspension was added with 5 µL of Annexin V-PE (G1512-1, Servicebio, China) and 5 µL of FITC (G1512-2, Servicebio, China), mixed gently and incubated at room temperature in the dark for 8–10 min. Finally, 400 µL of pre-cooled 1 × Binding Buffer was added, mixed gently, and analyzed within 1 h by flow cytometry or fluorescence microscopy.

### Statistic

Data analysis was conducted using GraphPad (version 8.0.2). After calculating mean and standard deviation of the data for each group, one-way ANOVA was used to compare the differences between the different groups and comparisons between each of the two groups were made using LSD method. We deemed results statistically significant when the *p*-value was below 0.05.

## Results

### Identification of DEGs and mitophagy-DEGs

**Fig. 2:**
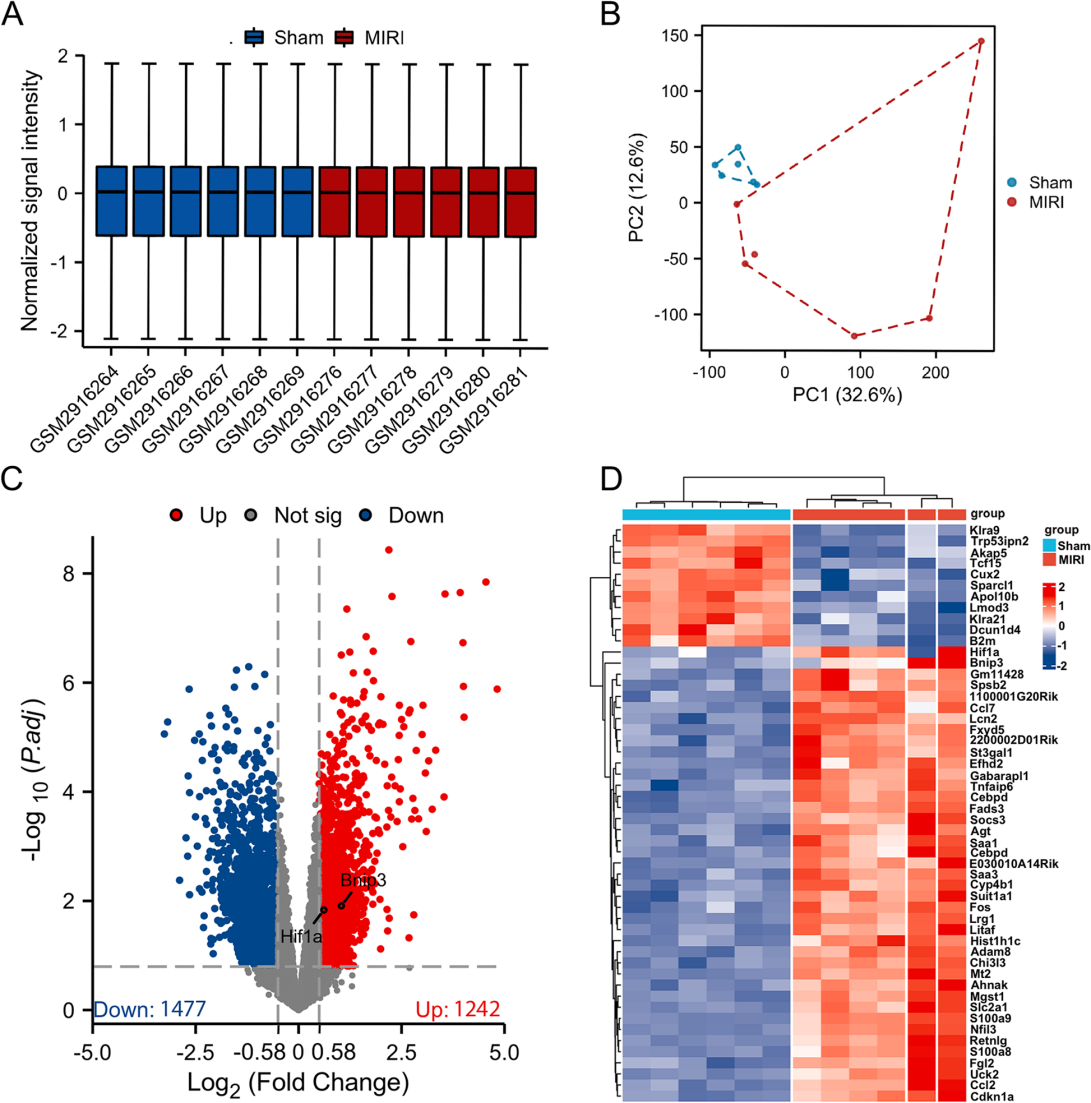
Data filtering and identification of DEGs in the myocardial ischemia–reperfusion injury tissue samples. **A** IQR boxplot and **B** PCA analysis show the data were well corrected. **C** Volcano plot of all genes. Red dots represented up-regulated genes and blue dots represented down-regulated genes. **D** Heat map for DEGs in myocardial ischemia–reperfusion injury and sham tissues. PCA, principal component analysis. DEGs, differentially expressed genes

**Fig. 3:**
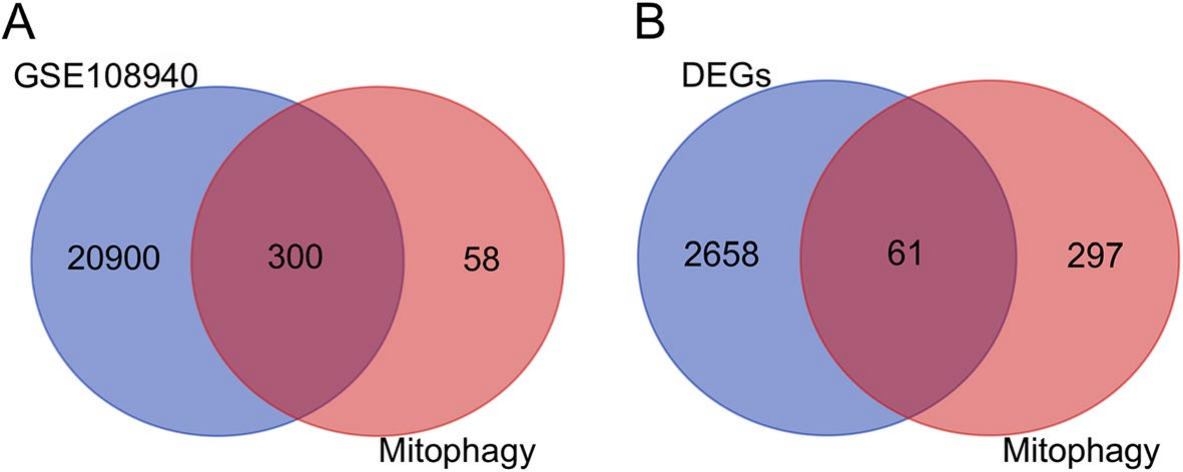
Identification of mitophagy-DEGs in the myocardial ischemia–reperfusion injury tissue. **A** The Venn diagram of gene expression profile data and mitophagy-related genes. **B** The Venn diagram of DEGs and mitophagy-related genes. Mitophagy-DEGs, mitophagy related with differentially expressed genes; DEGs, differentially expressed genes

GSE108940 from GEO database was analyzed for this study. A total of 6 normal myocardial tissues and 6 myocardial ischemia–reperfusion injury tissues were included for analysis. Firstly, the datasets were validated. The PCA results demonstrated high data reproducibility and minimal within-group differences (Fig. 2A, B). Then, DEGs were analyzed using the online analysis tool GEO2R in GEO. After screening with | log_2_FC|> 0.58 and *P.adj* < 0.05, 1242 upregulated and 1477 downregulated genes were determined for MIRI tissues (Table 2). A log2 fold change (log2FC) threshold of 0.58 signifies an increase or decrease in expression levels by 50%.In tightly regulated systems, this threshold enables the capture of such pivotal variations.A lower threshold also enhances the sensitivity of the analysis, detecting smaller yet potentially biologically relevant changes.The volcano plot (Fig. 2C) and heat map (Fig. 2D) displayed all the DEGs. Subsequently, we performed an intersection analysis between DEGs and 358 mitophagy-related genes, identifying 61 mitophagy-DEGs (Fig. 3), comprising 30 upregulated and 31 downregulated genes (Table 3).

**Table 2:** Top thirty DEGs in myocardial ischemia–reperfusion injury (GSE108940)

Gene Symbol	LogFC	*P*.Value	Level
Chi3l3	5.35631987	1.4587E-08	up
Saa3	5.00622591	5.0007E-08	up
Retnlg	4.82237652	1.3134E-06	up
Mt2	4.54967286	1.4343E-08	up
Saa1	4.01848555	4.2943E-06	up
S100a8	4.00365247	1.1722E-06	up
Lcn2	3.99205676	1.8572E-07	up
S100a9	3.9247197	2.2392E-08	up
Adam8	3.55634485	2.3765E-08	up
Clec4d	3.53286423	0.00012439	up
Mmp8	3.32903866	1.7316E-05	up
Inhbb	3.24501742	0.00020926	up
Cxcl5	3.15866983	2.7147E-05	up
Cxcl1	3.10000481	0.00053463	up
Ch25h	3.07821451	4.5374E-05	up
Gm129	-2.4028879	0.00012274	down
Gbp4	-2.4052287	0.00598493	down
C4bp-ps1	-2.4119188	8.7139E-06	down
Zc3h8	-2.5033922	0.00028753	down
Btnl9	-2.5255227	1.1414E-05	down
Pkd2l2	-2.6472027	0.00010386	down
Rbm20	-2.6473857	0.00769908	down
Apol10b	-2.6573319	1.3182E-06	down
H2-Aa	-2.6816034	5.1371E-05	down
Ccdc141	-2.6938	0.00151436	down
Il15	-2.7336388	0.00069808	down
Gbp5	-2.8896188	0.00417906	down
Klra21	-3.1796132	5.26E-06	down
Helt	-3.2595925	8.7353E-06	down

**Table 3:** Mitophagy differentially expressed genes of MIRI

Symbol	LogFC	*P* Value	Level
Hspa1a	2.34257	7.77929E-05	up
Naa16	1.35780	0.002450393	up
Eif4a2	1.26890	9.56336E-05	up
Arih1	1.25228	8.30998E-05	up
Tuba1c	1.22517	0.000948914	up
Plscr1	1.16994	0.005702184	up
Bnip3	1.03843	0.012300336	up
Map1lc3b	1.03651	9.44731E-05	up
Sqstm1	1.03311	1.38669E-05	up
Eif2s1	0.98593	0.004890184	up
Pfkp	0.88192	0.048458602	up
Hsp90aa1	0.87751	0.002539133	up
Gabarapl1	0.84094	1.75896E-06	up
Atg5	0.82046	0.001266914	up
App	0.77847	0.021625159	up
Rplp0	0.77566	9.32554E-05	up
Rps27a	0.77524	6.29236E-05	up
Rps18	0.77273	0.000204349	up
Eif4a1	0.75525	0.002231037	up
Phgdh	0.75521	0.012495311	up
Chchd3	0.71329	0.001689938	up
Clint1	0.70475	0.003045162	up
Rpl12	0.68515	6.3021E-05	up
Ubxn6	0.66411	0.00119764	up
Hnrnpd	0.64619	0.002031479	up
Ptrh2	0.62318	0.008095097	up
Hif1a	0.61290	0.014674576	up
Pi4k2a	0.60666	0.025377198	up
Rps3	0.59655	5.60036E-05	up
Rps15a	0.59197	5.29615E-05	up
Rnf41	-0.61349	0.03576	down
Prkdc	-0.63025	0.01446	down
Clec16a	-0.64129	0.03889	down
Mapk14	-0.64376	0.00078	down
Eef2	-0.64806	0.04214	down
Fancc	-0.67518	0.01101	down
Hspa9	-0.72484	0.00277	down
H19	-0.73408	0.03687	down
Idh2	-0.74966	0.01282	down
Fus	-0.75939	0.00995	down
Eif2ak2	-0.76473	0.01934	down
Prpf8	-0.77042	0.01652	down
Huwe1	-0.79147	0.03988	down
Acin1	-0.83472	0.04600	down
Hspd1	-0.90435	0.00106	down
Mon2	-1.04925	0.01875	down
Bcl2	-1.06266	0.00013	down
Atp6v1a	-1.09452	0.03538	down
Mavs	-1.09496	0.03245	down
Cnot4	-1.13399	0.01756	down
Csnk2a1	-1.14173	0.00280	down
Abcb10	-1.22277	0.03133	down
Abce1	-1.24841	0.01777	down
Prkaa2	-1.41382	0.04701	down
Vps13d	-1.44020	0.00406	down
Fbxw7	-1.49497	0.00733	down
Plec	-1.56802	0.01112	down
Atg14	-1.85089	0.01023	down
Vps35	-1.88905	0.00600	down
Tfrc	-1.94790	0.00003	down
Dsp	-2.03580	0.01381	down

### DEGs functional enrichment analysis

**Fig. 4:**
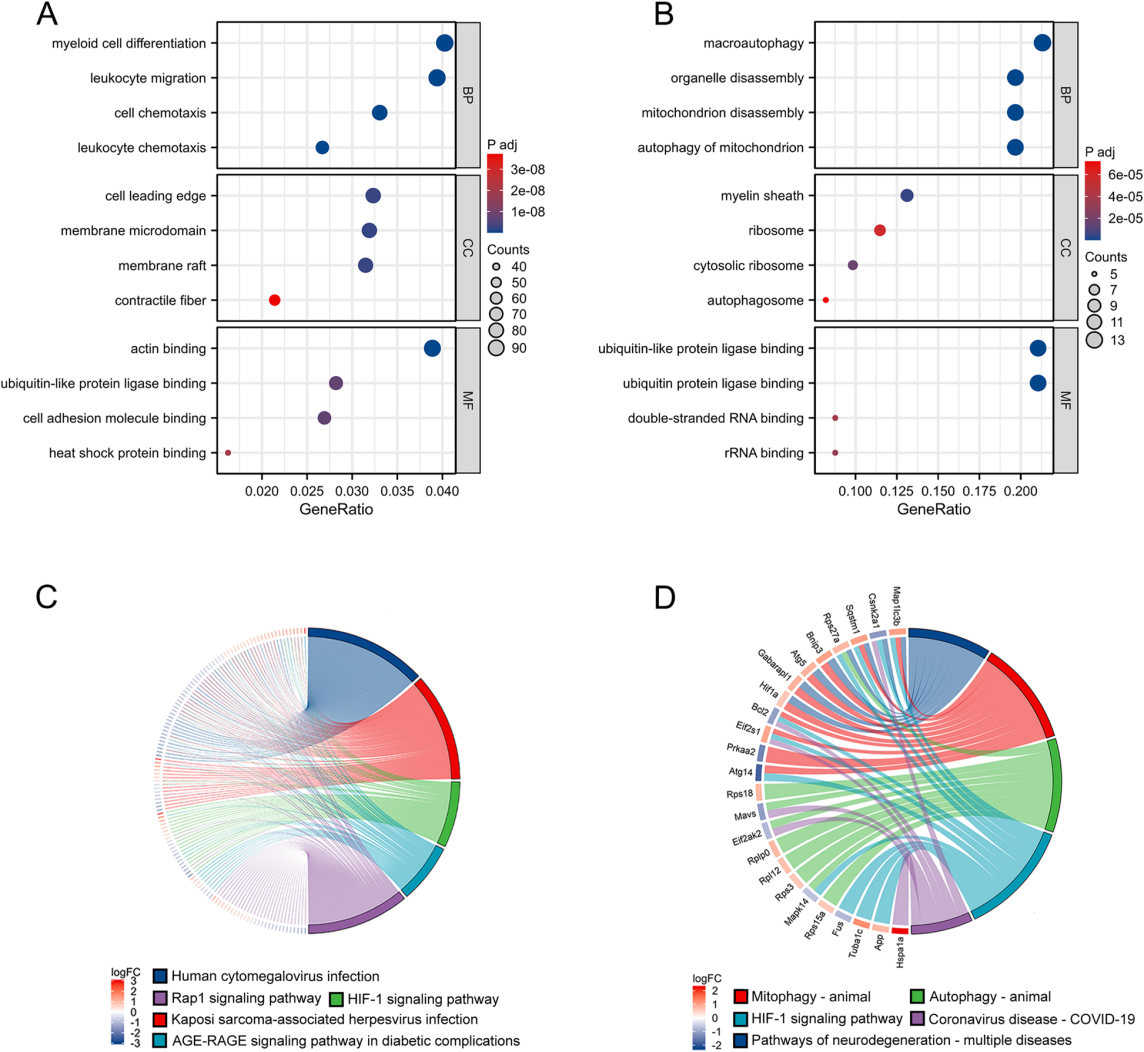
GO and KEGG enrichment analyses of DEGs. **A**, **B** Bubble plot of biological process, cellular component and molecular function of all DEGs and 61 mitophagy-DEGs GO analysis: The position of the bubbles indicates the proportion of significant genes associated with specific biological processes, cellular components, or molecular functions, the size of the bubbles reflects the number of significant genes, and the gradation of color denotes the level of significance post-*P*-value adjustment. **C** KEGG analysis of all DEGs. **D** KEGG analysis of 61 mitophagy-DEGs

Initially, enrichment analysis of the DEGs was conducted using GO and KEGG. GO BP analysis revealed significant enrichments in muscle tissue development, cell chemotaxis, myeloid cell differentiation, leukocyte chemotaxis, and wound healing. Regarding CC, the DEGs were explicitly associated with the apical part of the cell, cell leading edge, membrane microdomain, and nuclear envelope. The MF analysis unveiled that the DEGs predominantly correlated with actin binding, interactions with ubiquitin-like protein ligases, binding to cell adhesion molecules, amide binding, and heat shock protein interactions (Fig. 4A). The KEGG pathway analysis revealed significant enrichments in the HIF-1 signaling pathway, MAPK signaling pathway, mTOR signaling pathway, PI3K-Akt signaling pathway, autophagy-animal and Phagosome (Fig. 4C).

### Validation and enrichment analysis of mitophagy-DEGs

To explore the biological features of mitophagy-DEGs, we conducted GO and KEGG pathway enrichment analyses employing the clusterProfiler package within the R software environment (Table 4). The GO enrichment analysis yielded results in three functional categories: BP, CC, and MF (Fig. 4B). Within the BP category, mitophagy-DEGs showed significant enrichment in macroautophagy, organelle disassembly, autophagy of mitochondrion, reactive oxygen species metabolic process, and mitochondrion disassembly. Regarding CC, Mitophagy-DEGs were predominantly associated with the myelin sheath, ribosome, autophagosome, cytosolic ribosome, and autophagosome. Regarding MF, mitophagy-DEGs exhibited significant enrichments in ubiquitin-like protein ligase binding, ubiquitin protein ligase binding [23], ATP hydrolysis activity, and p53 binding. The KEGG pathway analysis revealed significant enrichments in mitophagy-animal, HIF-1 signaling pathway, NOD-like receptor signaling pathway, RIG-I-like receptor signaling pathway (Fig. 4D).

**Table 4:** Analysis of GO and KEGG enrichment of Mitophagy-DEGs

Term	Description	*P* Value	GeneID
GO: BP	autophagy	7.14143E-12	Bcl2/Map1lc3b/Fbxw7/Rnf41/Sqstm1/Huwe1/Bnip3/Atg5/Gabarapl1/Vps13d/Prkaa2/Hif1a/Ubxn6/Clec16a/Atg14
GO: BP	macroautophagy	1.1887E-12	Map1lc3b/Rnf41/Sqstm1/Huwe1/Bnip3/Atg5/Gabarapl1/Vps13dPrkaa2/Hif1a/Ubxn6/Clec16a/Atg14
GO: BP	organelle disassembly	3.78713E-15	Map1lc3b/Fbxw7/Rnf41/Sqstm1/Huwe1/Bnip3/Atg5/Gabarapl1/Vps13d/Hif1a/Clec16a/Atg14
GO: BP	response to extracellular stimulus	4.78674E-09	Bcl2/Map1lc3b/Eif2s1/Eif2ak2/Mapk14/Atg5/Gabarapl1/Tfrc/Prkaa2/Plec/Clec16a/Atg14
GO: BP	myeloid cell differentiation	9.23385E-09	Prkdc/Plscr1/Fbxw7/Rnf41/Cnot4/Mapk14/Hspa1a/Tfrc/Hspa9/Abcb10/Hif1a/App
GO: CC	myelin sheath	2.54497E-06	Hspd1/Bcl2/Phgdh/Atp6v1a/Rps27a/Hsp90aa1/Hspa9/Plec
GO: CC	ribosome	3.24078E-05	Rps18/Rplp0/Rpl12/Rps27a/Rps3/Eef2/Rps15a
GO: CC	early endosome	0.000120211	Hspd1/Vps35/Pi4k2a/Tfrc/Mon2/Ubxn6/App
GO: CC	cytosolic ribosome	9.54183E-06	Rps18/Rplp0/Rpl12/Rps27a/Rps3/Rps15a
GO: CC	autophagosome	4.1545E-05	Map1lc3b/Sqstm1/Atg5/Gabarapl1/Atg14
GO: MF	ubiquitin protein ligase binding	2.85494E-10	Hspd1/Bcl2/Arih1/Map1lc3b/Fbxw7/Sqstm1/Rps27a/Gabarapl1/Hspa1a/Hsp90aa1/Hspa9/Hif1a
GO: MF	ubiquitin-like protein ligase binding	2.85494E-10	Hspd1/Bcl2/Arih1/Map1lc3b/Fbxw7/Sqstm1/Rps27a/Gabarapl1/Hspa1a/Hsp90aa1/Hspa9/Hif1a
GO: MF	ATP hydrolysis activity	3.78331E-05	Hspd1/Abce1/Hspa1a/Hsp90aa1/Hspa9/Abcb10/Eif4a2
GO: MF	structural constituent of ribosome	3.78331E-05	Rps18/Rplp0/Rpl12/Rps27a/Rps3/Rps15a
GO: MF	rRNA binding	3.30564E-05	Rps18/Rplp0/Rpl12/Rps3/Eef2
KEEG	Mitophagy—animal	1.44545E-09	Map1lc3b/Csnk2a1/Sqstm1/Rps27a/Bnip3/Atg5/Gabarapl1/Hif1a
KEEG	NOD-like receptor signaling pathway	9.20304E-05	Bcl2/Map1lc3b/Mavs/Mapk14/Atg5/Gabarapl1/Hsp90aa1
KEEG	Ribosome	0.00027538	Rps18/Rplp0/Rpl12/Rps27a/Rps3/Rps15a
KEEG	HIF-1 signaling pathway	0.002645133	Pfkp/Bcl2/Tfrc/Hif1a
KEEG	RIG-I-like receptor signaling pathway	0.005385165	Mavs/Mapk14/Atg5

### Identification of key genes of Mitophagy-DEGs

**Fig. 5:**
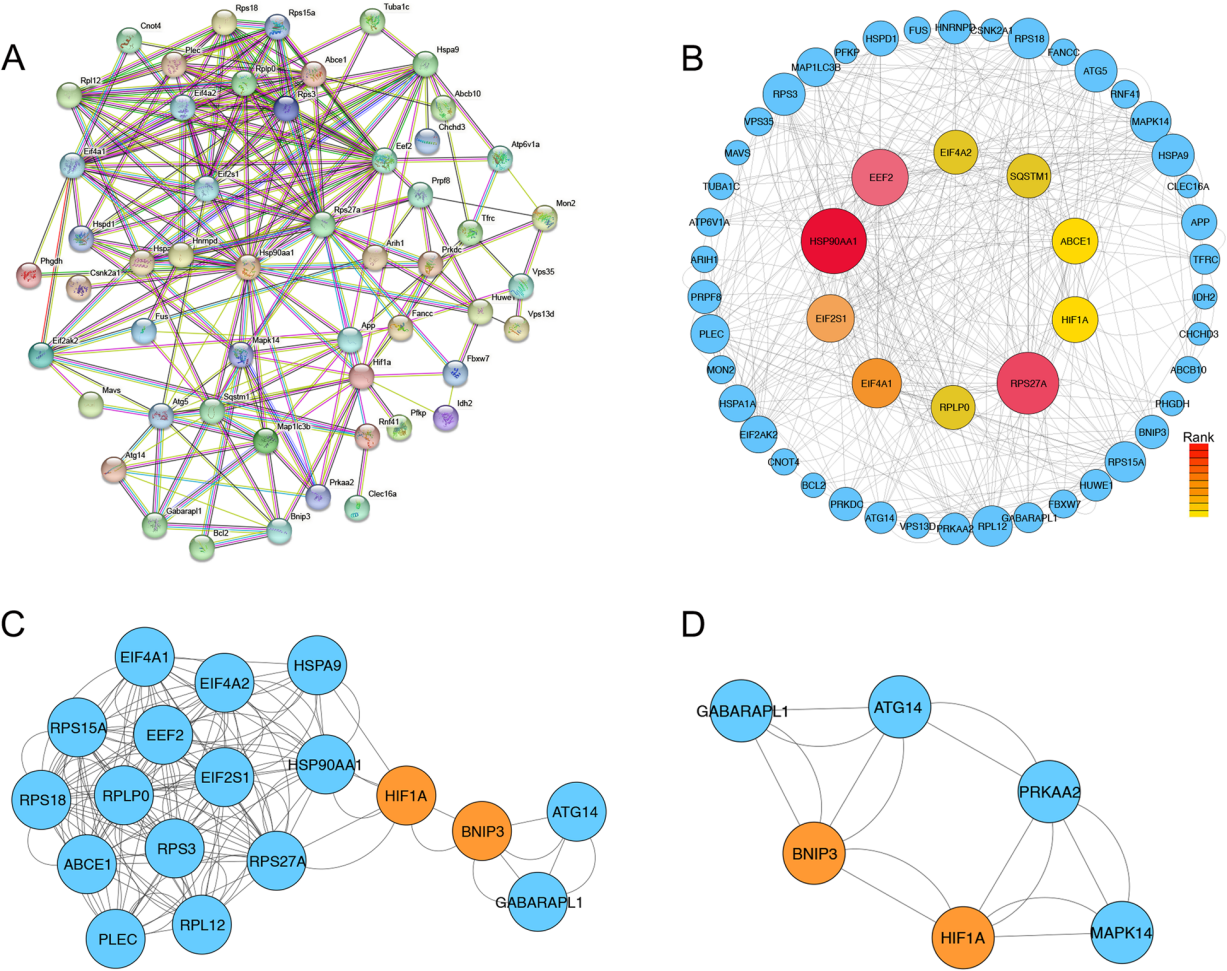
PPI network analysis of hub genes. **A** PPI network.from STRING database. **B** PPI network circles represented proteins and lines represented interactions between proteins. Ten hub genes were in colour. The red depth means the degree of importance. **C** Results scored 9.647 using the MCODE plugin in Cytoscape. **D** Results scored 5.4 using the MCODE plugin in Cytoscape; PPI, protein–protein interaction

The mitophagy-DEGs were uploaded to STRING website to obtain the PPI network (Fig. 5A). The top 7 hub genes, including HSP90AA1, RPS27A, EEF2, EIF4A1, EIF2S1, HIF-1α and BNIP3 were identified using cytoHubba (Fig. 5B, Table 5). Next, MCODE was employed to analyze PPI network.MCODE (Molecular Complex Detection) is primarily utilized for identifying and scoring functional modules or complexes within protein–protein interaction (PPI) networks, taking into account key parameters such as the weighted degree of nodes, the density of the complex, and node connectivity. Higher scores in subnetworks indicate a greater degree of protein interconnectivity, signifying a functionally more cohesive protein complex. Analysis of 61 proteins through MCODE resulted in 4 subnetworks with a maximum score of 10, and MCODE 1, with a score of 9.647, comprised of HSP90AA1, HIF-1α, BNIP3, ATG14, GABARAPL1, HSPA9, EIF4A1, EIF4A2, EEF2, EIF2S1, RPS15A, RPLP0, RPS3, RPS27A, RPS18, ABCE1, PLEC and RPL12 (Fig. 5C). MCODE 2, with a score of 5.4, consisted of HIF-1α, BNIP3, MAPK14, PRKAA2, ATG14 and GABARAPL1 (Fig. 5D). MCODE 3 and MCODE 4 scored 1.6 and 1.5, respectively. The higher scores of MCODE 1 and MCODE 2 further elucidate the close association between HIF-1α and BNIP3, laying a theoretical foundation for subsequent in-depth exploration of their connection with MIRI.

**Table 5:** Details of Seven hub genes

Gene Symbol	Degree	Full names
HSP90AA1	6	heat shock protein 90 alpha family class A member 1
RPS27A	5	ribosomal protein S27a
EEF2	5	eukaryotic elongation factor 2
EIF4A1	5	eukaryotic translation initiation factor 4A1
EIF2S1	4	eukaryotic translation initiation factor 2 subunit 1
HIF-1α	2	hypoxia-inducible factor 1 alpha
BNIP3	2	Bcl-2/adenovirus E1B 19 kDa protein-interacting protein 3

### GSEA analysis HIF-1α and BNIP3

**Fig. 6:**
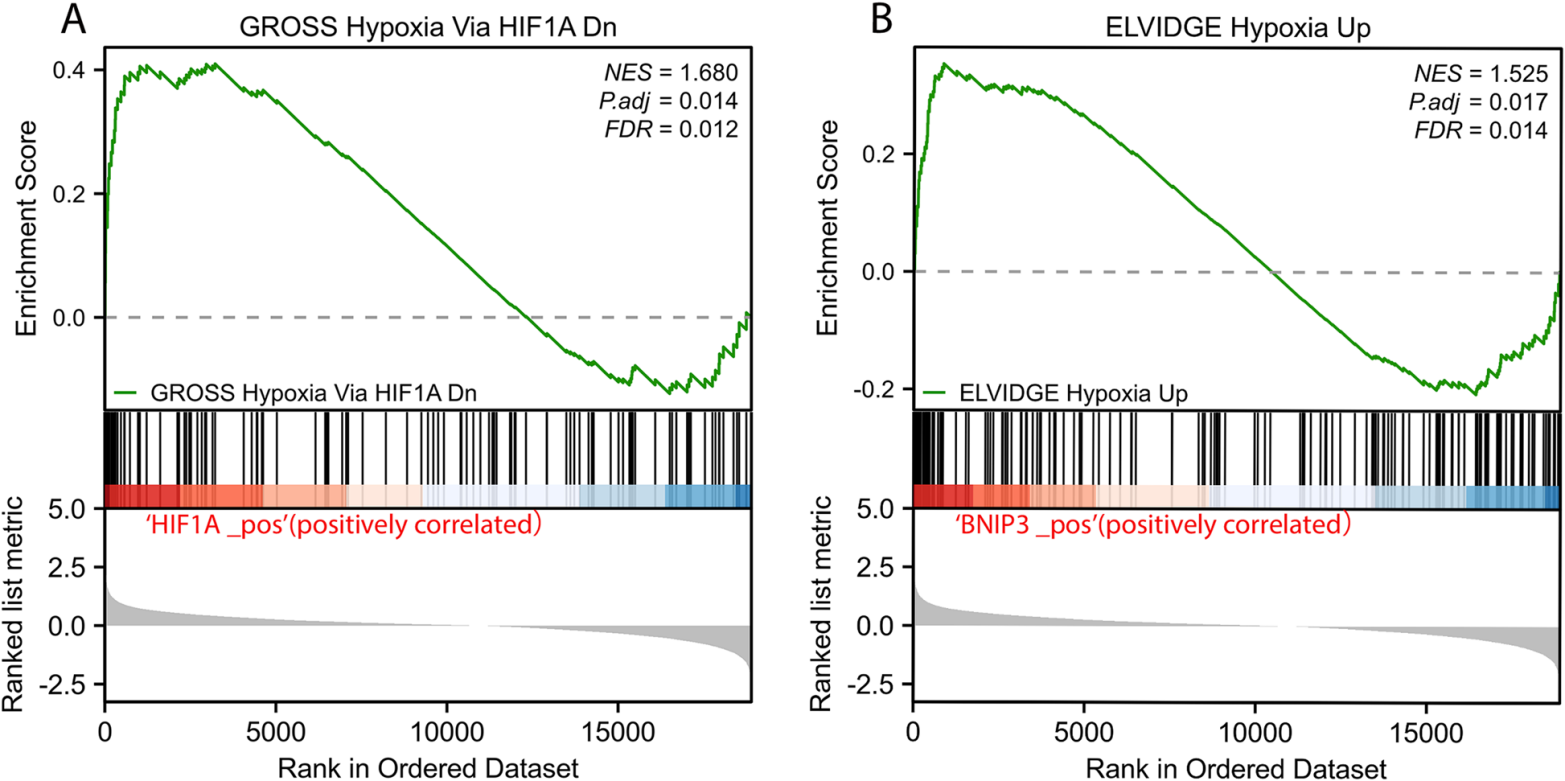
GSEA analysis of hub genes. **A**, **B** Represent the pathways about function enrichment of HIF-1α and BNIP3; GSEA, gene set enrichment analysis

The pivotal genes HIF-1α and BNIP3 were subjected to enrichment analysis using GSEA software with *FDR* < 0.01. The pathways associated with each hub gene were determined by analyzing their expression profiles by KEGG pathway database. The analysis revealed that the enriched pathways were predominantly related to GROSS Hypoxia Via HIF1A Dn and ELVIDGE Hypoxia Up (Fig. 6A, B).

### Successfully established MIRI rat model

**Fig. 7:**
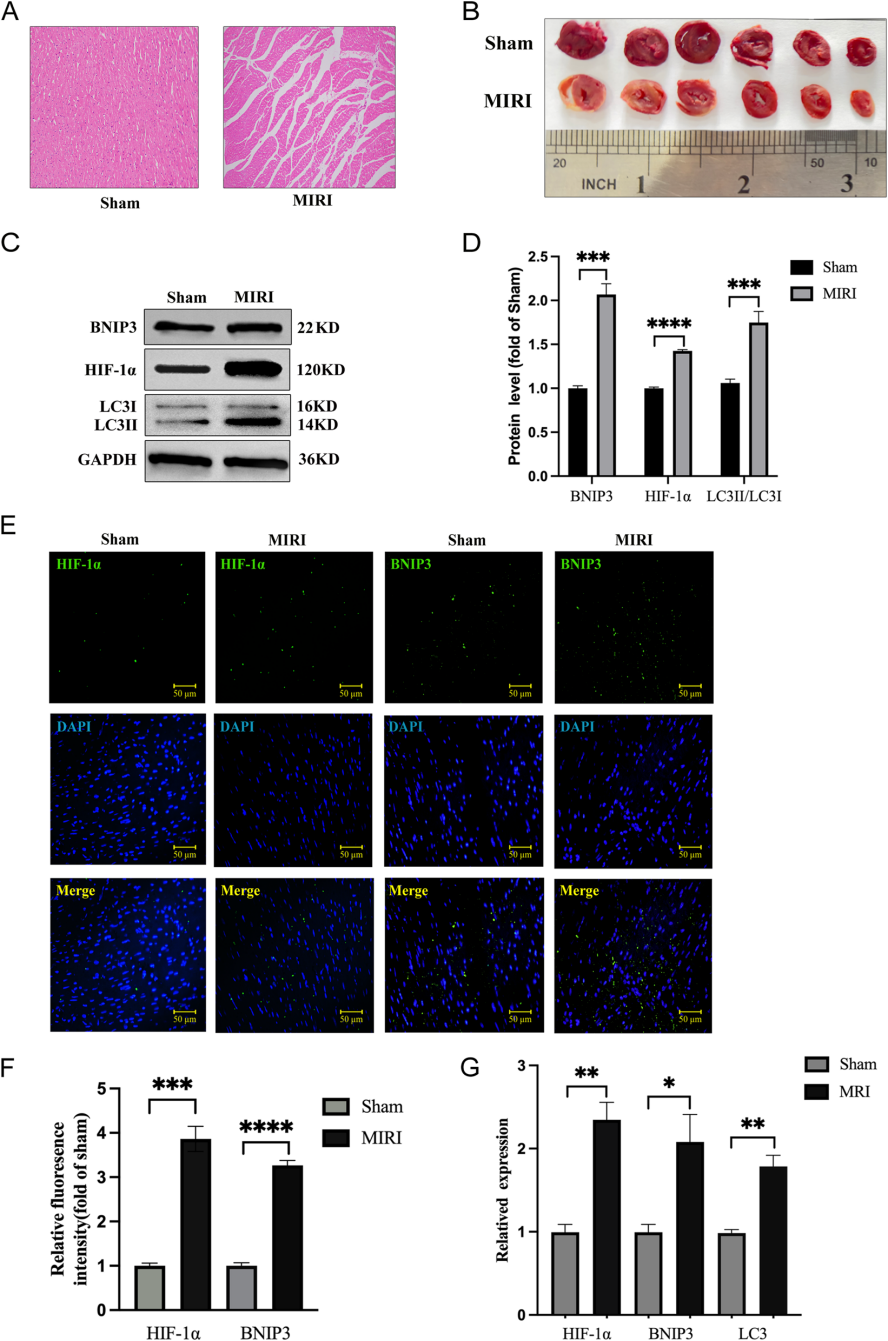
Validation of the expression of hub genes. **A** The results of HE: Sham group myocardial cells were neatly arranged in striated patterns.MIRI group exhibited disorganized myocardial cell arrangement, with instances of myocardial rupture, nuclear deformation. **B** The results of TTC: The myocardial tissue in Sham group typically shows a uniform red coloration. MIRI group myocardial tissue appear pale or white. These areas represent necrotic or infarcted tissue. **C**, **D** Western blot analysis of HIF-1α, BNIP3, LC3I and LC3II protein levels in Sham and MIRI group (The experiments were repeated three times). **E**, **F** Immunofluorescence analysis of HIF-1α and BNIP3 in Sham and MIRI group. **G** QPCR results for mRNA levels of hub genes HIF-1α and BNIP3; ***P < 0.001, ****P < 0.0001

HE staining showed that the shape, size and structure of cardiomyocytes were normal in the Sham group [24], while in the MIRI group, large areas of cardiomyocytes were dead, with obvious cell degeneration and hypertrophy; myocardial fibers were disorganized, and the survival rate of cardiomyocytes was reduced, suggesting that MIRI model was effectively established in rats [25] (Fig. 7A). For TTC staining, compared to Sham group, the infarct area of the MIRI group increased (*P* < 0.05) (Fig. 7B).

### Expression levels of HIF-1α, BNIP3 and LC3 in myocardial tissue

Comparatively, in MIRI group, expression of HIF-1α and BNIP3 proteins increased significantly (*P* < 0.05) and the ratio of LC3II to LC3I increased compared to Sham group (*P* < 0.05), indicating an increased expression of mitophagy related proteins following MIRI (Fig. 7C, D). The results show that IRI significantly promotes the expression of mitochondrial autophagy-related proteins, which may be associated with the activation of the HIF-1α/BNIP3 pathway.

### Immunofluorescence of HIF-1α and BNIP3

HIF-1α and BNIP3 expression were well verified by immunofluorescence staining. In contrast to sham group, there was a notable elevation in HIF-1α expression within the MIRI group (*P* < 0.05), and notable disparity was observed in the expression of BNIP3 (*P* < 0.05). The immunofluorescent staining signals, represented in green, clearly indicate the heightened expression levels and fluorescence intensity of HIF-1α and BNIP3 within the MIRI group [26]. The observed differences were considered statistically significant when compared to the Sham group (Fig. 7E, F).

### Validation of findings via qRT-PCR

The qPCR was conducted using six MIRI and six paired Sham tissue samples to validate the mRNA expression of HIF-1α, BNIP3 and LC3. The obtained results align with our expectations, revealing a significant upregulation of HIF-1α, BNIP3 and LC3 in the MIRI group (*p* < 0.05), in stark contrast to their lower expression levels in the Sham group (Fig. 7G).

### Expression levels of HIF-1α, BNIP3 and LC3 in H9C2

**Fig. 8:**
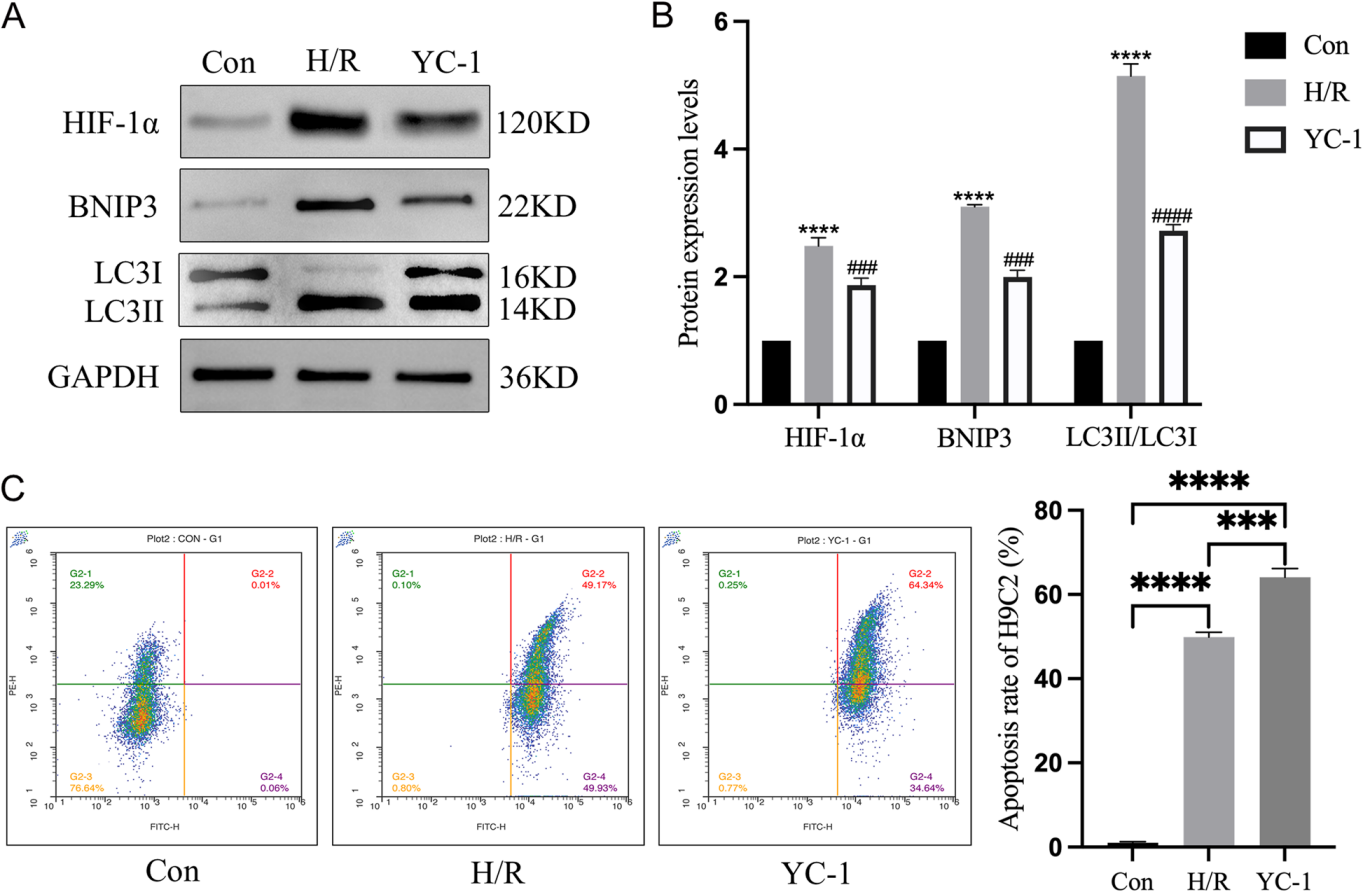
Investigation into the Mechanism of HIF-1α and BNIP3 in MIRI. **A**, **B** Western blot analysis of HIF-1α, BNIP3, LC3I and LC3II protein levels in Con, H/R and YC-1 group; Compared with Con group,****P < 0.001,compared with H/R group,^###^*P* < 0.01. **C** Apoptosis rate of H9C2 cardiomyocytes in each group; ***P < 0.001,****P < 0.0001

Compared to the control group, the expression levels of HIF-1α and BNIP3 proteins, as well as the LC3II/LC3I ratio, were significantly elevated in the H/R group (*P* < 0.01), suggesting an induction of mitochondrial autophagy following H/R.Indicating that the expression of HIF-1α could promote the expression of BNIP3. In contrast, the YC-1 group exhibited a significant reduction in the expression levels of HIF-1α, BNIP3, and the LC3II/LC3I ratio compared to the H/R group (*P* < 0.001) (Fig. 8A, B).

### Apoptotic rate in Rat H9C2 Cardiomyocytes

Compared with the control group, the rate of cardiomyocyte apoptosis in the H/R group was significantly increased (*P* < 0.01) (Fig. 8C); compared with the H/R group, the apoptosis rate in the YC-1 group further increased (*P* < 0.01) (Fig. 8C). This indicates that inhibiting the expression of HIF-1α and BNIP3, thereby countering mitochondrial autophagy, is actually detrimental to H9C2 cardiomyocytes, leading to further myocardial cell damage and apoptosis.

### Study limitation

Despite the deployment of various bioinformatics analysis and statistical methodologies to investigate myocardial ischemia–reperfusion injury, it is imperative to acknowledge the inherent limitations of our study. Firstly, the limited sample size and single-sex animal models may have overlooked certain genes potentially implicated in myocardial ischemia–reperfusion injury. Secondly, species differences warrant careful consideration, with future experiments needed to further explore interspecies correlations. Thirdly, our study's database relies solely on microarray data from GEO, RNA-seq, and Genecards. Future research endeavors will aim to amalgamate data from multiple sources. Lastly, the model used in this study to investigate myocardial ischemia–reperfusion injury carries specific constraints.

## Discussion

Our analysis indicates that mitophagy is activated during myocardial ischemia–reperfusion injury. Additionally, we identified and analyzed relevant hub genes, providing new directions for treating myocardial ischemia–reperfusion injury. From the GSE108940 database, we obtained 2719 DEGs, including 1242 upregulated genes and 1477 downregulated genes. Through KEGG enrichment analysis, we discovered the significant role of mitophagy in MIRI. Subsequently, we obtained 61 intersecting genes from intersection of DEGs and mitophagy database. Through enrichment analysis, these genes were primarily associated with oxidative stress, the HIF-1 signaling pathway, and mitophagy, thereby obtaining hub genes. The seven highest-scoring genes include HSP90AA1, RPS27A, EEF2, EIF4A1, EIF2S1, HIF-1α and BNIP3. However, we discovered through functional clustering using the MCODE plugin that there is a close relationship between HIF-1α and BNIP3, so we included BNIP3 in our study. Analyzing hub genes with GSEA software further substantiates the involvement of mitophagy in MIRI, particularly HIF-1α/BNIP3 signaling pathway. Finally, experimental validation was conducted to confirm the expression of pivotal genes.

In this study, we identified the top 7 hub genes. HSP90AA1, or Hsp90α, belongs to the family of HSP90. It plays essential physiological and regulatory roles within cells. Studies have shown that HSP90AA1 is significantly protective in myocardial ischemia–reperfusion injury [27]. HSP90AA1 is involved in regulating cellular autophagy, thereby protecting myocardial cells from MIRI damage [28]. Research has demonstrated that HSP90AA1 can modulate the expression of autophagy-related proteins, including cathepsin and LC3, thereby promoting autophagy [29]. Moreover, HSP90AA1 exerts antioxidant effects by neutralizing free radical production and reducing oxidative stress-induced damage to myocardial cells [30]. Studies have shown that HSP90AA1 can enhance the activity of antioxidant enzymes, incorporating enzymes such as superoxide dismutase (SOD) and glutathione peroxidase (GPx), consequently resulting in a decrease in levels of oxidative stress [30, 31].

RPS27A, a component of the ribosome, participates in the regulation of protein synthesis [32, 33]. Firstly, RPS27A participates in the recognition and initiation of mitophagy signals. Research has identified an interaction between RPS27A and mitochondrial damage markers, such as Parkin and PINK1 [34, 35]. Upon mitochondrial damage, cells tag the damaged mitochondria through specific signaling pathways and initiate mitophagy [36]. Secondly, RPS27A interacts with proteins on the autophagosomal membrane, including LC3 and autophagy adapter proteins such as sequestosome 1 (SQSTM1, also known as p62) [35, 37]. This interaction further facilitates the association of mitochondria with the autophagosomal membrane, promoting the engulfment and degradation of mitochondria [38, 39].

The post-translational modified factor eukaryotic elongation factor 2 (EEF2) serves as a critical regulatory factor in ischemia–reperfusion injury [40]. Firstly, EEF2 regulates protein synthesis via its phosphorylation state [41]. Under normal conditions, unphosphorylated EEF2 facilitates ribosomal translocation, enabling smooth protein synthesis [42]. However, during the process of ischemia–reperfusion injury, the activity of the phosphorylating enzyme EEF2 kinase significantly increases, resulting in excessive phosphorylation of EEF2. This phosphorylation state inhibits EEF2 function, leading to suppressed protein synthesis [43]. Secondly, EEF2 is also implicated in cell death. Studies have shown that the phosphorylation state of EEF2 plays a critical regulatory role in ischemia–reperfusion injury [44]. Excessive phosphorylation of EEF2 leads to inhibited protein synthesis and enhanced cell death pathways, including apoptosis and autophagy [45]. Studies have shown that inhibiting the phosphorylation of EEF2 can attenuate IRI and reduce cell death [46, 47].

EIF4A1 is a translation initiation factor that interacts with proteins involved in mitophagy, including Parkin and PINK1 [48]. These interactions contribute to initiating mitophagy and promoting selective degradation of damaged mitochondria. Furthermore, EIF4A1 can regulate gene expression associated with mitophagy, thereby influencing the process [49].

EIF2S1 is a transfer ribonucleic acid (tRNA)-binding protein that participates in the regulation of translation initiation [50, 51]. In the context of myocardial ischemia, EIF2S1 undergoes phosphorylation and activation by PERK (protein kinase R-like endoplasmic reticulum kinase) [52]. This activation inhibits the initiation of protein translation, reducing the demand for oxygen and nutrients and enhancing the process of mitophagy to mitigate cellular damage [53]. However, during the early reperfusion phase, the accumulation of damaged proteins necessitates clearance through degradation. Phosphorylation of EIF2S1 also modulates cellular stress responses and inflammatory reactions, further influencing the regulation of mitophagy [54, 55].

**Fig. 9:**
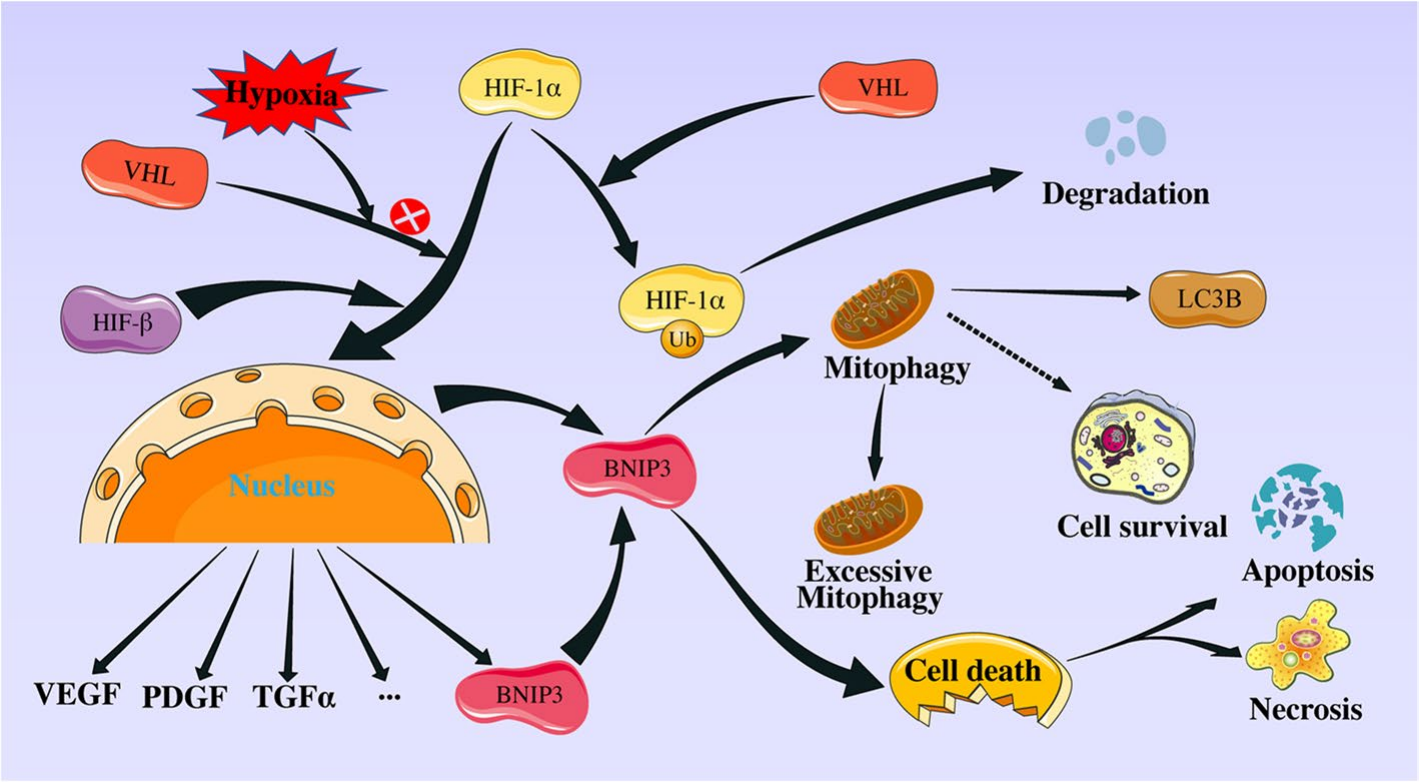
Schematic representation of the mechanism of mitophagy and HIF-1α/BNIP3 pathway in MIRI

Meanwhile, we proposed the following mechanism of action based on the data (Fig. 9). When ischemia–reperfusion injury occurred, it induced localized hypoxia and led to the production of superoxide within the corneal epithelium. Under hypoxic conditions, oxygen availability decreases, leading to stabilization and accumulation of HIF-1α. After stabilization, HIF-1α migrates into the nucleus and associates with HIF-1β to form an active HIF-1 complex [56]. Once this active HIF-1 complex is formed, it binds to specific hypoxia response elements (HREs) located within the promoter regions of target genes, thereby regulating the transcription of BNIP3 [56, 57]. HIF-1-regulated BNIP3 interacted with LC3 on the phagophore membrane, marking the damaged mitochondria for sequestration and subsequent autophagosomal engulfment. This interaction is mediated by LC3-interacting regions (LIRs) present in BNIP3 [58, 59]. BNIP3 also recruits autophagy receptors, such as NIX (BNIP3L), to enhance mitophagy efficiency. Hypoxia, a potent inducer of BNIP3 expression, activates HIF-1, which directly binds to the BNIP3 promoter, promoting its transcription. In addition, AMP-activated protein kinase (AMPK) activation, triggered by cellular energy depletion, phosphorylates and activates BNIP3, enhancing its pro-mitophagy function. Moreover, BNIP3 can be regulated by upstream kinases, such as Akt and GSK-3β, which modulate its activity and subcellular localization. The BCL2 family of proteins, comprising anti-apoptotic and pro-apoptotic members, influences BNIP3-mediated mitophagy [60]. BNIP3 contains a BH3 domain that enables its interaction with anti-apoptotic BCL2 family members, such as BCL2 and BCL-XL [61]. This interaction releases the pro-apoptotic protein Beclin-1, initiating autophagy. Moreover, BNIP3 can promote mitochondrial outer membrane permeabilization (MOMP), leading to cytochrome c release and apoptotic cell death, highlighting its dual role in autophagy and apoptosis [62, 63].

Currently, there is a growing body of evidence indicating that I/R can disrupt the process of mitophagy [64], leading to further cytotoxic damage and potentially resulting in cell death. Research has additionally demonstrated a direct correlation between the extent of myocardial ischemia and autophagic processes in myocardial I/R [45]. These viewpoints align precisely with the results of our bioinformatics analysis. It’s worth noting that there is still a debate over the primary mechanism of “autophagic cell death”, whether it involves excessive mitophagy or the extensive accumulation of autophagosomes [65]. A new consensus is emerging that the changes induced by cellular damage align with the formation and initiation of autophagosomes in myocardial cells, contributing to the process of cell death [66]. Nevertheless, as the molecular mechanisms underlying autophagy’s dual function in I/R remain incompletely elucidated, further investigations are warranted in numerous instances to ascertain whether autophagy can exert protective or deleterious effects [67, 68].

Furthermore, the HIF-1α/BNIP3-mediated regulation of mitophagy that we have discovered provides new insights into potential therapeutic targets for myocardial ischemia–reperfusion injury [69]. A growing body of scholars has begun to direct their attention towards the role of autophagy in myocardial protection, holding the promise of unveiling novel therapeutic strategies for MIRI [70]. A multitude of preclinical investigations have been undertaken to evaluate the pharmacological modulation of autophagic flux as a potent strategy against MIRI, yielding noteworthy outcomes. It has been discovered that many intervention measures, such as resveratrol, allicin, resveratrol, and hydrogen-rich saline, can protect the heart from the impacts of IRI by enhancing autophagy levels [71]. Furthermore, certain medications such as ryanodine, JNK inhibitors, berberine, and trimetazidine have shown potential in heart protection by either inhibiting autophagy or enhancing mitochondrial repair. Additionally, distinct non-coding RNAs that target autophagy have emerged as pivotal players in MIRI [72]. Consequently, the ongoing quest to pinpoint precise cellular mechanisms, sustain optimal autophagic processes, and curtail excessive autophagy represents a central therapeutic endeavor, offering a promising outlook for future strategies in addressing MIRI and enhancing cardiac protection.

## Conclusion

In summary, the current study is the first study that comprehensively explores the involvement of mitophagy and the HIF-1α/BNIP3 pathway in MIRI through integrated bioinformatics analysis, which has provided new insights into the pathophysiological mechanisms of treating ischemia–reperfusion injury. Our research results indicate that the dual regulation of mitophagy levels is associated with IRI, which may offer valuable clues for exploring the therapeutic mechanisms of myocardial ischemia–reperfusion injury. Furthermore, this study suggests that the seven mitophagy-signature genes (HSP90AA1, RPS27A, EEF2, EIF4A1, EIF2S1, HIF-1α, BNIP3) may serve not only as potential biomarkers for myocardial ischemia–reperfusion but also as potential targets for future research and Paving the way for novel therapeutic approaches targeting myocardial ischemia–reperfusion injury.

### Supplementary Information

**Supplementary Material 1. **

## Data Availability

The analysis in this study utilized publicly available datasets. The data can be accessed at the following links: https://www.ncbi.nlm.nih.gov/geo/query/acc.cgi?acc=GSE108940 and https://www.genecards.org/Search/Keyword?queryString=mitophagy.

## References

[1] Chen Z, Wu D, Li L, Chen L (2016). Apelin/APJ System: A Novel Therapeutic Target for Myocardial Ischemia/Reperfusion Injury. DNA Cell Biol.

[2] Lahiri S, Roy A, Baby SM, Hoshi T, Semenza GL, Prabhakar NR (2006). Oxygen sensing in the body. Prog Biophys Mol Biol.

[3] Guo Y (2017). Role of HIF-1a in regulating autophagic cell survival during cerebral ischemia reperfusion in rats. Oncotarget.

[4] Koeppen M, Lee JW, Seo SW, Brodsky KS, Kreth S, Yang IV, Buttrick PM, Eckle T, Eltzschig HK (2018). Hypoxia-inducible factor 2-alpha-dependent induction of amphiregulin dampens myocardial ischemia-reperfusion injury. Nat Commun.

[5] Li X, Guo L, Wang J, Yang X (2023). Pro-fibrotic and apoptotic activities of circARAP1 in myocardial ischemia-reperfusion injury. Eur J Med Res.

[6] Yuan X, Lee JW, Bowser JL, Neudecker V, Sridhar S, Eltzschig HK (2018). Targeting hypoxia signaling for perioperative organ injury. Anesth Analg.

[7] Tan Z, Dong F, Wu L, Feng Y, Zhang M, Zhang F (2023). Transcutaneous Electrical Nerve Stimulation (TENS) alleviates brain ischemic injury by regulating neuronal oxidative stress, pyroptosis, and mitophagy. Mediators Inflamm.

[8] Fu Z-J, Wang Z-Y, Xu L, Chen X-H, Li X-X, Liao W-T, Ma H-K, Jiang M-D, Xu T-T, Xu J (2020). HIF-1α-BNIP3-mediated mitophagy in tubular cells protects against renal ischemia/reperfusion injury. Redox Biology.

[9] Xia J-Y, Chen C, Lin Q, Cui J, Wan J, Li Y, Li D (2023). Role of mitophagy in myocardial ischemia/reperfusion injury and chinese medicine treatment. Chin J Integr Med.

[10] Choi ME (2020). Autophagy in kidney disease. Annu Rev Physiol.

[11] Moulin S, Arnaud C, Bouyon S, Pépin J-L, Godin-Ribuot D, Belaidi E (2020). Curcumin prevents chronic intermittent hypoxia-induced myocardial injury. Ther Adv Chronic Dis.

[12] Wang J, Toan S, Zhou H (2020). New insights into the role of mitochondria in cardiac microvascular ischemia/reperfusion injury. Angiogenesis.

[13] Jiang X, Wu D, Jiang Z, Ling W, Qian G (2021). Protective effect of nicorandil on cardiac microvascular injury: role of mitochondrial integrity. Oxid Med Cell Longev.

[14] Han Y, Jin G, Pan M, Fang Z, Lu D, Cai W, Xu C (2022). Integrated bioinformatics and validation of lncRNA-mediated ceRNA network in myocardial ischemia/reperfusion injury. J Immunol Res.

[15] Yang M, Linn BS, Zhang Y, Ren J (2019). Mitophagy and mitochondrial integrity in cardiac ischemia-reperfusion injury. Biochim Biophys Acta.

[16] Yang X, Pan W, Xu G, Chen L (2020). Mitophagy: A crucial modulator in the pathogenesis of chronic diseases. Clin Chim Acta.

[17] Jin Q, Li R, Hu N, Xin T, Zhu P, Hu S, Ma S, Zhu H, Ren J, Zhou H (2018). DUSP1 alleviates cardiac ischemia/reperfusion injury by suppressing the Mff-required mitochondrial fission and Bnip3-related mitophagy via the JNK pathways. Redox Biol.

[18] Wang X, Zhang J, Xiu C, Yang J, Liu Y, Lei Y (2023). Whole-transcriptome sequencing analysis reveal mechanisms of Yiqi Huoxue Yangyin (YHY) decoction in ameliorating D-gal-induced cardiac aging. Aging (Albany NY).

[19] Ferreira AP, Rodrigues FS, Della-Pace ID, Mota BC, Oliveira SM, de Campos Velho Gewehr C, Bobinski F, de Oliveira CV, Brum JS, Oliveira MS (2014). HOE-140, an antagonist of B2 receptor, protects against memory deficits and brain damage induced by moderate lateral fluid percussion injury in mice. Psychopharmacology (Berl).

[20] Dong H, Zhang C, Shi D, Xiao X, Chen X, Zeng Y, Li X, Xie R (2023). Ferroptosis related genes participate in the pathogenesis of spinal cord injury via HIF-1 signaling pathway. Brain Res Bull.

[21] Shen S, He F, Cheng C, Xu B, Sheng J (2021). Uric acid aggravates myocardial ischemia-reperfusion injury via ROS/NLRP3 pyroptosis pathway. Biomed Pharmacother.

[22] Han G, Xu J, Chen Q, Xia X, Liu H, Kong B (2022). Improving the solubility of myofibrillar proteins in water by destroying and suppressing myosin molecular assembly via glycation. Food Chem.

[23] He S, He L, Yan F, Li J, Liao X, Ling M, Jing R, Pan L (2022). Identification of hub genes associated with acute kidney injury induced by renal ischemia-reperfusion injury in mice. Front Physiol.

[24] Zhang Y, Liu D, Hu H, Zhang P, Xie R, Cui W (2019). HIF-1α/BNIP3 signaling pathway-induced-autophagy plays protective role during myocardial ischemia-reperfusion injury. Biomed Pharmacother.

[25] Xiong Y, Xia Y, Deng J, Yan X, Ke J, Zhan J, Zhang Z, Wang Y (2020). Direct peritoneal resuscitation with pyruvate protects the spinal cord and induces autophagy via regulating PHD2 in a rat model of spinal cord ischemia-reperfusion injury. Oxid Med Cell Longev.

[26] Yang L, Wu J, Xie P, Yu J, Li X, Wang J, Zheng H (2019). Sevoflurane postconditioning alleviates hypoxia-reoxygenation injury of cardiomyocytes by promoting mitochondrial autophagy through the HIF-1/BNIP3 signaling pathway. PeerJ.

[27] Xiao X, Wang W, Li Y, Yang D, Li X, Shen C, Liu Y, Ke X, Guo S, Guo Z (2018). HSP90AA1-mediated autophagy promotes drug resistance in osteosarcoma. J Exp Clin Cancer Res.

[28] Zhu WS, Guo W, Zhu JN, Tang CM, Fu YH, Lin QX, Tan N, Shan ZX (2016). Hsp90aa1: a novel target gene of miR-1 in cardiac ischemia/reperfusion injury. Sci Rep.

[29] Yang S, Nie T, She H, Tao K, Lu F, Hu Y, Huang L, Zhu L, Feng D, He D (2023). Regulation of TFEB nuclear localization by HSP90AA1 promotes autophagy and longevity. Autophagy.

[30] Pan Z, Bao Y, Hu M, Zhu Y, Tan C, Fan L, Yu H, Wang A, Cui J, Sun G (2023). Role of NAT10-mediated ac4C-modified HSP90AA1 RNA acetylation in ER stress-mediated metastasis and lenvatinib resistance in hepatocellular carcinoma. Cell Death Discov.

[31] Toraih EA, Alrefai HG, Hussein MH, Helal GM, Khashana MS, Fawzy MS (2019). Overexpression of heat shock protein HSP90AA1 and translocase of the outer mitochondrial membrane TOM34 in HCV-induced hepatocellular carcinoma: A pilot study. Clin Biochem.

[32] Luo J, Zhao H, Chen L, Liu M (2023). Multifaceted functions of RPS27a: An unconventional ribosomal protein. J Cell Physiol.

[33] Suzuki M, Tezuka K, Handa T, Sato R, Takeuchi H, Takao M, Tano M, Uchida Y (2022). Upregulation of ribosome complexes at the blood-brain barrier in Alzheimer's disease patients. J Cereb Blood Flow Metab.

[34] Ahmed MM, Tazyeen S, Haque S, Alsulimani A, Ali R, Sajad M, Alam A, Ali S, Bagabir HA, Bagabir RA (2021). Network-based approach and IVI methodologies, a combined data investigation identified probable key genes in cardiovascular disease and chronic kidney disease. Front Cardiovasc Med.

[35] Hu Z, Liu R, Hu H, Ding X, Ji Y, Li G, Wang Y, Xie S, Liu X, Ding Z (2022). Potential biomarkers of acute myocardial infarction based on co-expression network analysis. Exp Ther Med.

[36] Bowles KR, Abraham SE, Brugada R, Zintz C, Comeaux J, Sorajja D, Tsubata S, Li H, Brandon L, Gibbs RA (2000). Construction of a high-resolution physical map of the chromosome 10q22-q23 dilated cardiomyopathy locus and analysis of candidate genes. Genomics.

[37] Zhang H, Ye J, Weng X, Liu F, He L, Zhou D, Liu Y (2015). Comparative transcriptome analysis reveals that the extracellular matrix receptor interaction contributes to the venous metastases of hepatocellular carcinoma. Cancer Genet.

[38] Nim HT, Dang L, Thiyagarajah H, Bakopoulos D, See M, Charitakis N, Sibbritt T, Eichenlaub MP, Archer SK, Fossat N (2021). A cis-regulatory-directed pipeline for the identification of genes involved in cardiac development and disease. Genome Biol.

[39] Olivieri JE, Dehghannasiri R, Wang PL, Jang S, de Morree A, Tan SY, Ming J, Ruohao WuA, Tabula Sapiens C, Quake SR (2021). RNA splicing programs define tissue compartments and cell types at single-cell resolution. Elife.

[40] Zhang C, Liu X, Zhang C, Li J, Guo W, Yan D, Yang C, Zhao J, Wu X, Shi J (2017). Phosphorylated eEF2 is SUMOylated and induces cardiomyocyte apoptosis during myocardial ischemia reperfusion. J Cardiol.

[41] Zhang C, Liu X, Miao J, Wang S, Wu L, Yan D, Li J, Guo W, Wu X, Shen A (2017). Heat shock protein 70 protects cardiomyocytes through suppressing SUMOylation and nucleus translocation of phosphorylated eukaryotic elongation factor 2 during myocardial ischemia and reperfusion. Apoptosis.

[42] Kim AS, Miller EJ, Wright TM, Li J, Qi D, Atsina K, Zaha V, Sakamoto K, Young LH (2011). A small molecule AMPK activator protects the heart against ischemia-reperfusion injury. J Mol Cell Cardiol.

[43] Ratchford SM, Bailey AN, Senesac HA, Hocker AD, Smolkowski K, Lantz BA, Jewett BA, Gilbert JS, Dreyer HC (2012). Proteins regulating cap-dependent translation are downregulated during total knee arthroplasty. Am J Physiol Regul Integr Comp Physiol.

[44] Crozier SJ, Vary TC, Kimball SR, Jefferson LS (2005). Cellular energy status modulates translational control mechanisms in ischemic-reperfused rat hearts. Am J Physiol Heart Circ Physiol.

[45] Yang Y, Lin X (2023). Potential relationship between autophagy and ferroptosis in myocardial ischemia/reperfusion injury. Genes Dis.

[46] Garcia L, O'Loghlen A, Martin ME, Burda J, Salinas M (2004). Does phosphorylation of eukaryotic elongation factor eEF2 regulate protein synthesis in ischemic preconditioning?. J Neurosci Res.

[47] DeGracia DJ (2004). Acute and persistent protein synthesis inhibition following cerebral reperfusion. J Neurosci Res.

[48] Kacal M, Zhang B, Hao Y, Norberg E, Vakifahmetoglu-Norberg H (2021). Quantitative proteomic analysis of temporal lysosomal proteome and the impact of the KFERQ-like motif and LAMP2A in lysosomal targeting. Autophagy.

[49] Qureshi AA, Khan DA, Mushtaq S, Ye SQ, Xiong M, Qureshi N (2018). delta-Tocotrienol feeding modulates gene expression of EIF2, mTOR, protein ubiquitination through multiple-signaling pathways in chronic hepatitis C patients. Lipids Health Dis.

[50] Yao RQ, Ren C, Xia ZF, Yao YM (2021). Organelle-specific autophagy in inflammatory diseases: a potential therapeutic target underlying the quality control of multiple organelles. Autophagy.

[51] Mijit M, Boner M, Cordova RA, Gampala S, Kpenu E, Klunk AJ, Zhang C, Kelley MR, Staschke KA, Fishel ML (2023). Activation of the integrated stress response (ISR) pathways in response to Ref-1 inhibition in human pancreatic cancer and its tumor microenvironment. Front Med (Lausanne).

[52] Sehrawat U, Haimov O, Weiss B, Tamarkin-Ben Harush A, Ashkenazi S, Plotnikov A, Noiman T, Leshkowitz D, Stelzer G, Dikstein R (2022). Inhibitors of eIF4G1-eIF1 uncover its regulatory role of ER/UPR stress-response genes independent of eIF2alpha-phosphorylation. Proc Natl Acad Sci U S A.

[53] Zhang X, Yuan Y, Jiang L, Zhang J, Gao J, Shen Z, Zheng Y, Deng T, Yan H, Li W (2014). Endoplasmic reticulum stress induced by tunicamycin and thapsigargin protects against transient ischemic brain injury: Involvement of PARK2-dependent mitophagy. Autophagy.

[54] Li Y, Shen Y, Xie M, Wang B, Wang T, Zeng J, Hua H, Yu J, Yang M (2022). LncRNAs LCETRL3 and LCETRL4 at chromosome 4q12 diminish EGFR-TKIs efficiency in NSCLC through stabilizing TDP43 and EIF2S1. Signal Transduct Target Ther.

[55] Lozano J, Rai A, Lees JG, Fang H, Claridge B, Lim SY, Greening DW (2022). Scalable generation of nanovesicles from human-induced pluripotent stem cells for cardiac repair. Int J Mol Sci.

[56] Tarnawski AS, Jones MK (2003). Inhibition of angiogenesis by NSAIDs: molecular mechanisms and clinical implications. J Mol Med (Berl).

[57] Zheng J, Chen P, Zhong J, Cheng Y, Chen H, He Y, Chen C (2021). HIF-1α in myocardial ischemia-reperfusion injury (Review). Mol Med Rep.

[58] Liu X-W, Lu M-K, Zhong H-T, Wang L-H, Fu Y-P (2019). Panax Notoginseng Saponins Attenuate Myocardial Ischemia-Reperfusion Injury Through the HIF-1α/BNIP3 Pathway of Autophagy. J Cardiovasc Pharmacol.

[59] Yang L, Xie P, Wu J, Yu J, Li X, Ma H, Yu T, Wang H, Ye J, Wang J (2020). Deferoxamine Treatment Combined With Sevoflurane Postconditioning Attenuates Myocardial Ischemia-Reperfusion Injury by Restoring HIF-1/BNIP3-Mediated Mitochondrial Autophagy in GK Rats. Front Pharmacol.

[60] Ma X, Godar RJ, Liu H, Diwan A (2012). Enhancing lysosome biogenesis attenuates BNIP3-induced cardiomyocyte death. Autophagy.

[61] Zhang Y-N, Pang Y-X, Liu D-W, Hu H-J, Xie R-Q, Cui W (2022). JMJD5 attenuates oxygen-glucose deprivation and reperfusion-induced injury in cardiomyocytes through regulation of HIF-1α-BNIP3. Kaohsiung J Med Sci.

[62] Zhang J, Han X, Chang J, Liu J, Liu Y, Wang H, Du F, Zeng X, Guo C (2022). Soluble RAGE attenuates myocardial I/R injuries via FoxO3-Bnip3 pathway. Cell Mol Life Sci.

[63] Kunimi H, Lee D, Ibuki M, Katada Y, Negishi K, Tsubota K, Kurihara T (2021). Inhibition of the HIF-1α/BNIP3 pathway has a retinal neuroprotective effect. FASEB J.

[64] Zhu N, Li J, Li Y, Zhang Y, Du Q, Hao P, Li J, Cao X, Li L (2020). Berberine Protects Against Simulated Ischemia/Reperfusion Injury-Induced H9C2 Cardiomyocytes Apoptosis In Vitro and Myocardial Ischemia/Reperfusion-Induced Apoptosis In Vivo by Regulating the Mitophagy-Mediated HIF-1α/BNIP3 Pathway. Front Pharmacol.

[65] Chen W, Wang J, Wang X, Chang P, Liang M (2022). Knockdown of hypoxia-inducible factor 1-alpha (HIF1α) interferes with angiopoietin-like protein 2 (ANGPTL2) to attenuate high glucose-triggered hypoxia/reoxygenation injury in cardiomyocytes. Bioengineered.

[66] Cai W, Xu D, Zeng C, Liao F, Li R, Lin Y, Zhao Y, Dong W, Wang Q, Yang H (2022). Modulating lysine crotonylation in cardiomyocytes improves myocardial outcomes. Circ Res.

[67] Chen L, Shi D, Guo M (2021). The roles of PKC-δ and PKC-ε in myocardial ischemia/reperfusion injury. Pharmacol Res.

[68] Zhang M, Lei Y-S, Meng X-W, Liu H-Y, Li L-G, Zhang J, Zhang J-X, Tao W-H, Peng K, Lin J (2021). Iguratimod alleviates myocardial ischemia/reperfusion injury through inhibiting inflammatory response induced by cardiac fibroblast pyroptosis via COX2/NLRP3 signaling pathway. Front Cell Dev Biol.

[69] Chen X-Y, Wang J-Q, Cheng S-J, Wang Y, Deng M-Y, Yu T, Wang H-Y, Zhou W-J (2021). Diazoxide post-conditioning activates the HIF-1/HRE pathway to induce myocardial protection in hypoxic/reoxygenated cardiomyocytes. Front Cardiovasc Med.

[70] Li X, Gui Z, Liu H, Qian S, Jia Y, Luo X (2021). Remifentanil pretreatment ameliorates H/R-induced cardiac microvascular endothelial cell dysfunction by regulating the PI3K/Akt/HIF-1α signaling pathway. Bioengineered.

[71] Peng K, Chen W-R, Xia F, Liu H, Meng X-W, Zhang J, Liu H-Y, Xia Z-Y, Ji F-H (2020). Dexmedetomidine post-treatment attenuates cardiac ischaemia/reperfusion injury by inhibiting apoptosis through HIF-1α signalling. J Cell Mol Med.

[72] Liu X-W, Lu M-K, Zhong H-T, Liu J-J, Fu Y-P (2021). Panax notoginseng saponins protect H9c2 cells from hypoxia-reoxygenation injury through the forkhead box O3a hypoxia-inducible factor-1 alpha cell signaling pathway. J Cardiovasc Pharmacol.

