# Supplementary figures and images for: Bioinformatics integration reveals key genes associated with mitophagy in myocardial ischemia-reperfusion injury
Source: BMC Cardiovasc Disord. 2024 Mar 27;24:183. doi: 10.1186/s12872-024-03834-x (PMC10967080; doi:10.1186/s12872-024-03834-x)

~190 kDa  
~130 kDa  
~100 kDa  
~90 kDa  
~58 kDa  
~42 kDa  
~38 kDa  
~30 kDa  
~20 kDa  
~13 kDa  
~8 kDa

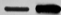

Supplement: Supplementary file 1 — Supplementary Material 1. [file 12872_2024_3834_MOESM1_ESM.zip › 1-Hif-1a myocardial tissue.pdf]

190 kDa-  
130 kDa-  
100 kDa-  
90 kDa-  
58 kDa-

42kDa-

38 kDa-

30 kDa-

20 kDa-

13 kDa-

8 kDa-

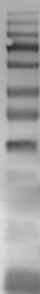

Supplement: Supplementary file 1 — Supplementary Material 1. [file 12872_2024_3834_MOESM1_ESM.zip › BNIP3-H9C2.pdf]

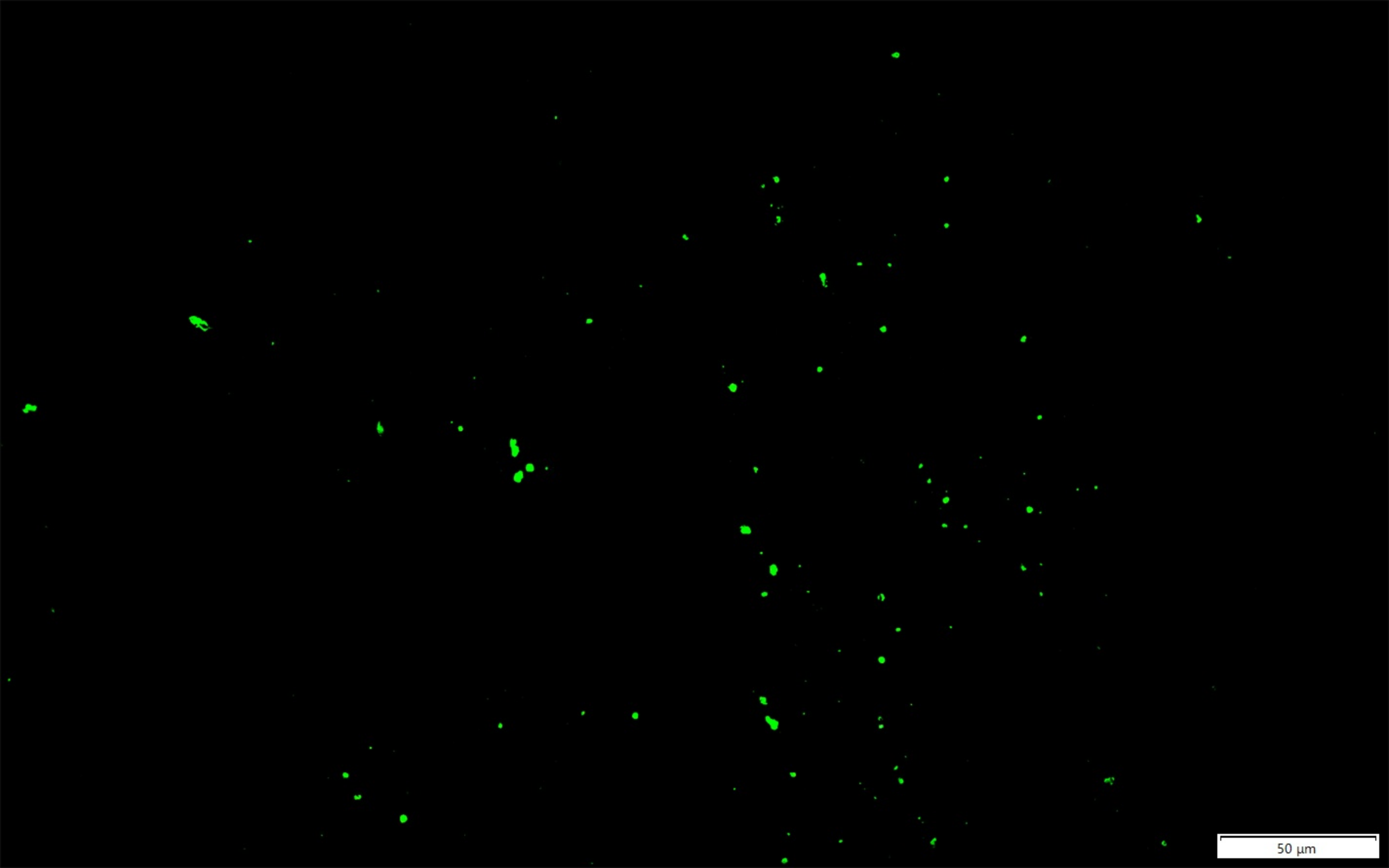

50 μm

Supplement: Supplementary file 1 — Supplementary Material 1. [file 12872_2024_3834_MOESM1_ESM.zip › BNIP3-MIRI-BNIP3.pdf]

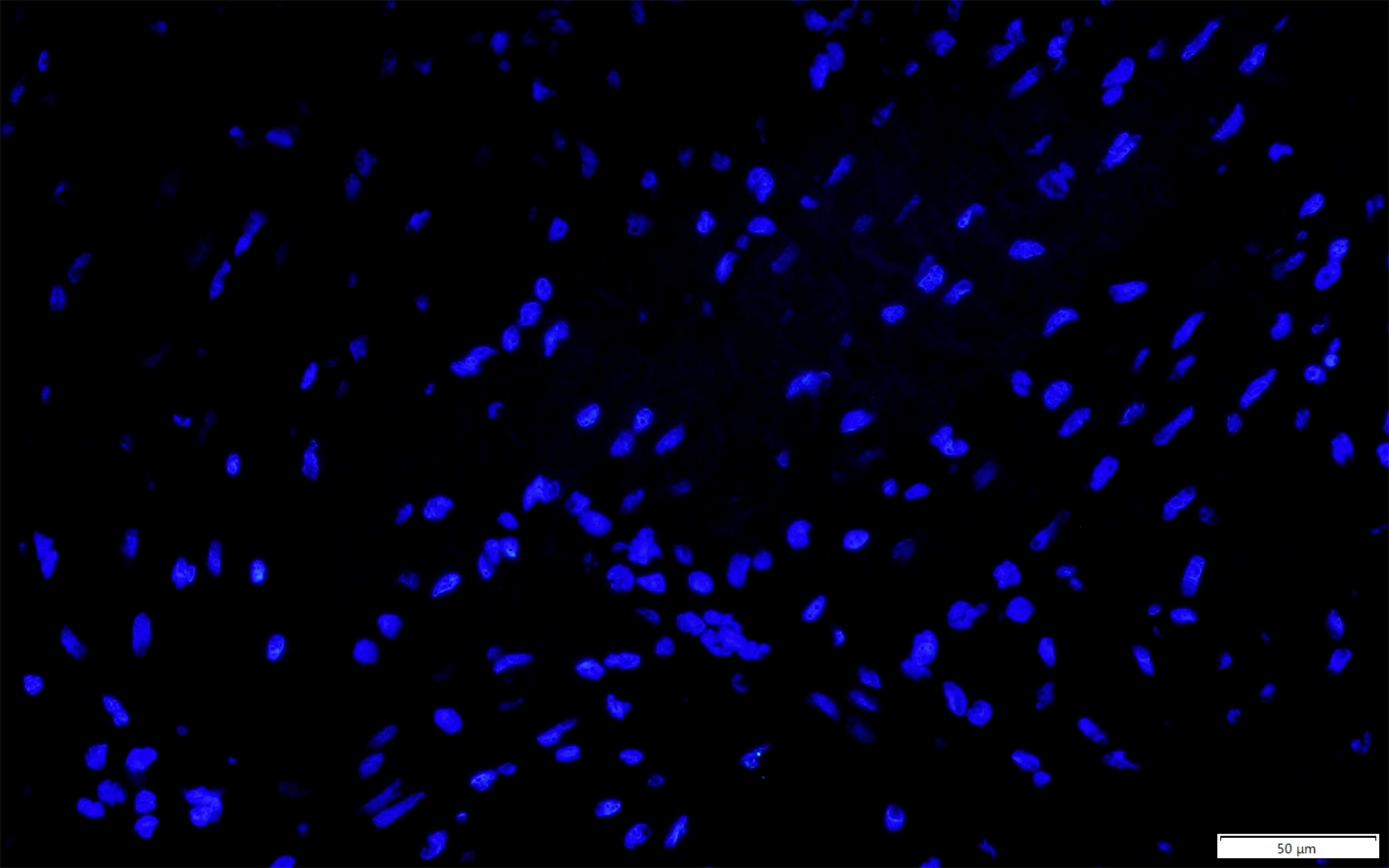

50 μm

Supplement: Supplementary file 1 — Supplementary Material 1. [file 12872_2024_3834_MOESM1_ESM.zip › BNIP3-MIRI-DAPI.pdf]

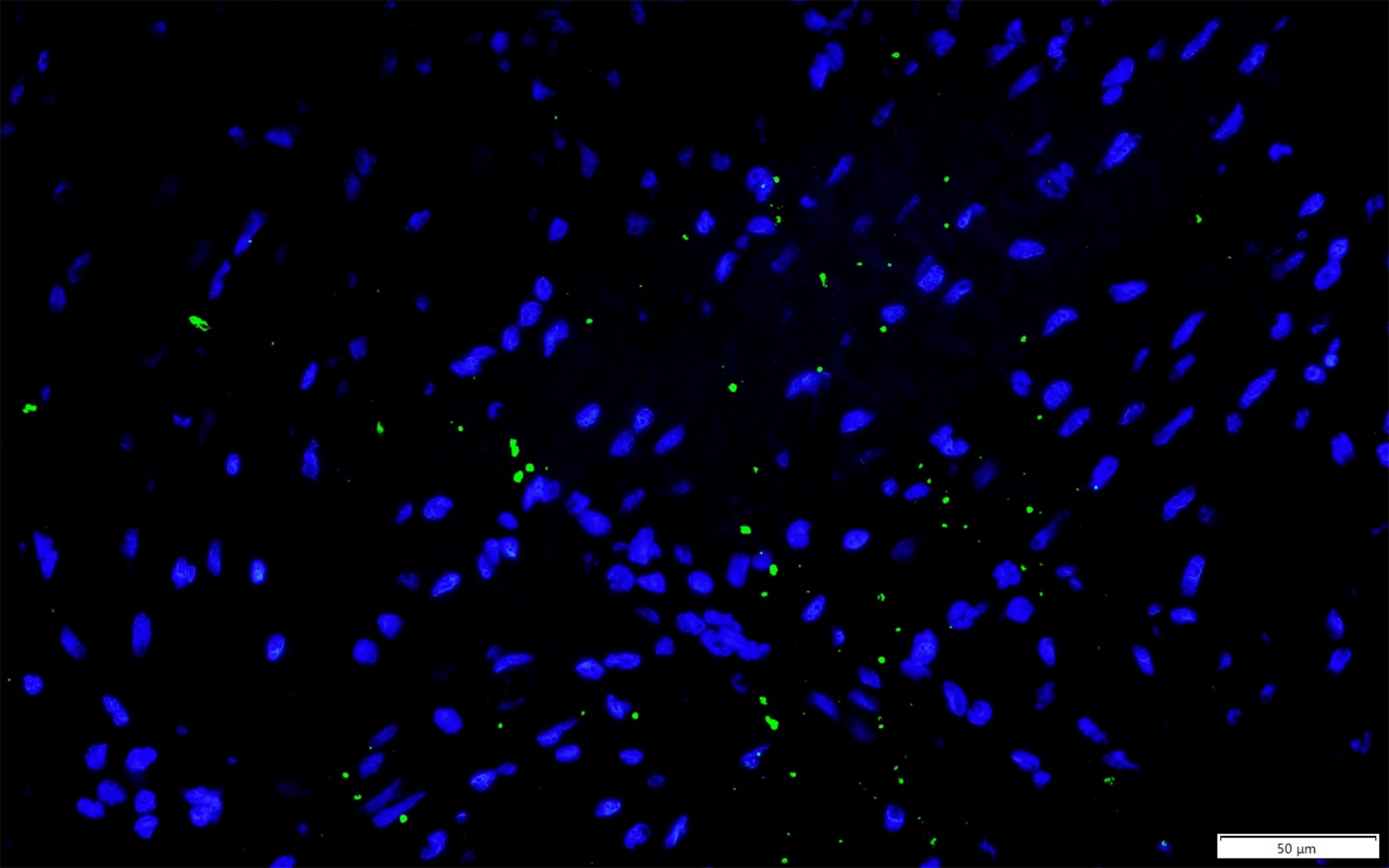

50  $\mu\text{m}$

Supplement: Supplementary file 1 — Supplementary Material 1. [file 12872_2024_3834_MOESM1_ESM.zip › BNIP3-MIRI-merge.pdf]

~200 kDa  
~140 kDa  
~110 kDa  
~90 kDa  
~68 kDa  
~55 kDa  
~40 kDa  
~30 kDa  
~20 kDa  
~13 kDa  
~8 kDa

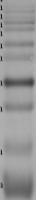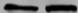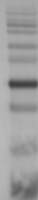

Supplement: Supplementary file 1 — Supplementary Material 1. [file 12872_2024_3834_MOESM1_ESM.zip › BNIP3-myocardial tissue.pdf]

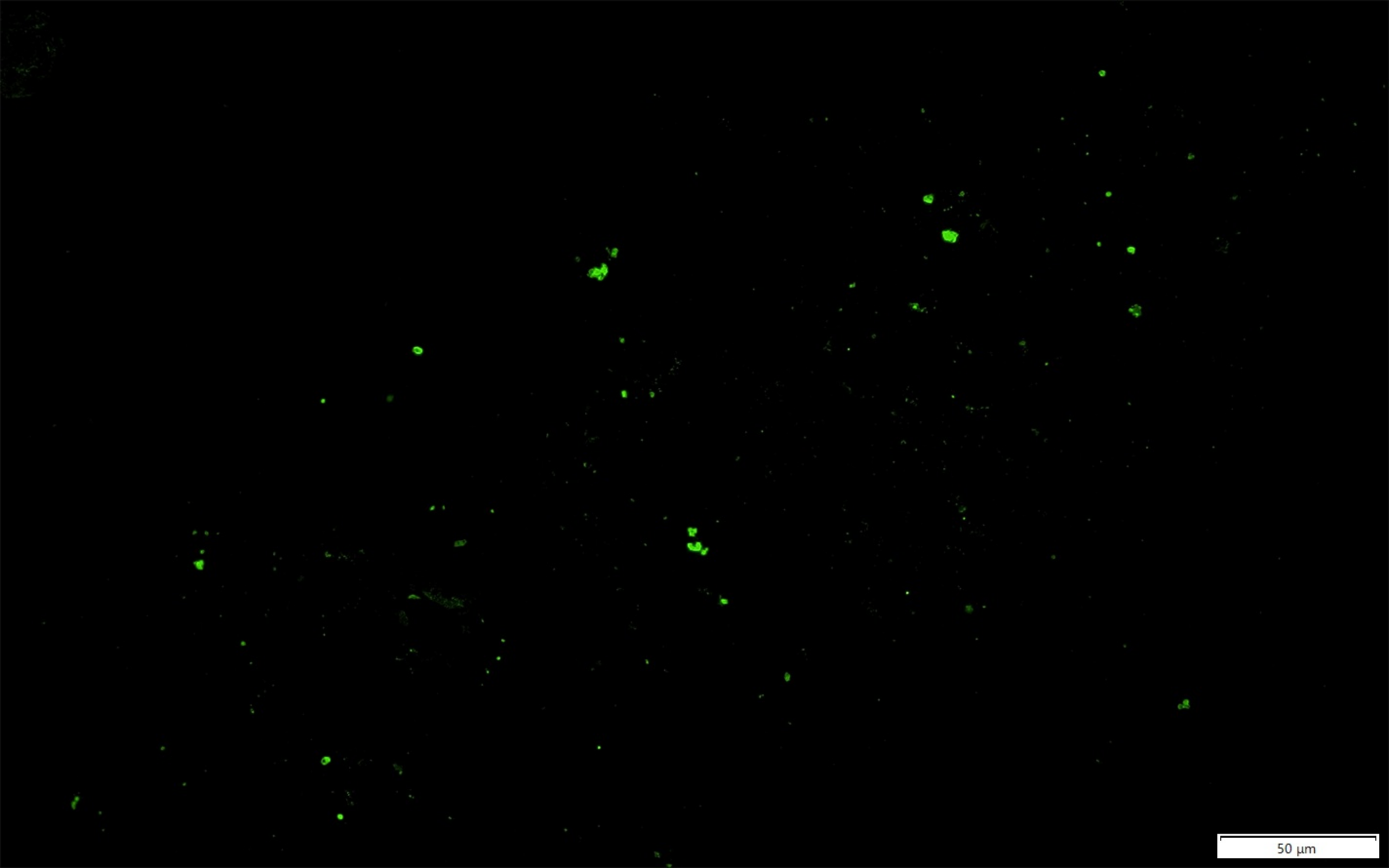

50 μm

Supplement: Supplementary file 1 — Supplementary Material 1. [file 12872_2024_3834_MOESM1_ESM.zip › BNIP3-sham-BNIP3.pdf]

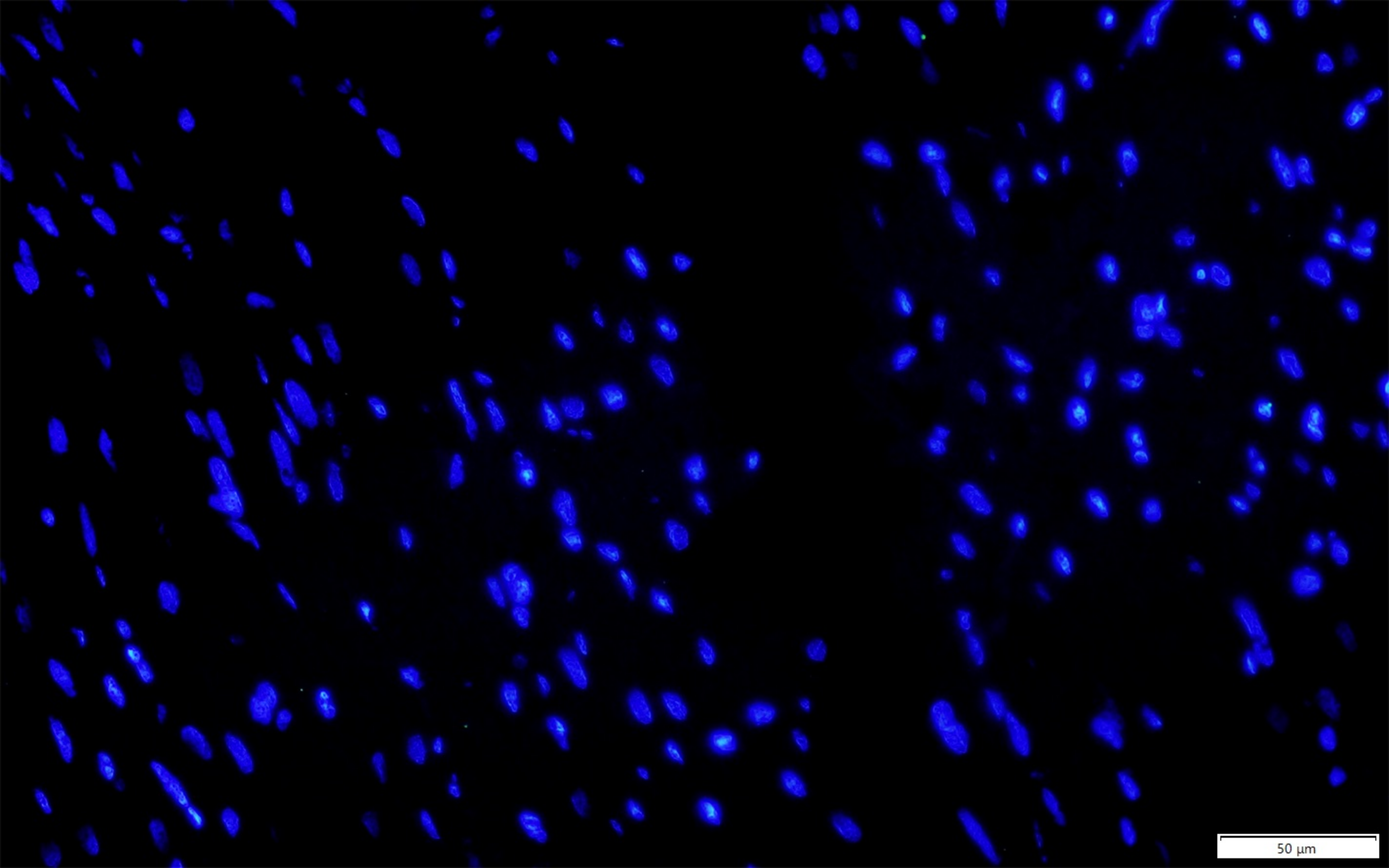

50 μm

Supplement: Supplementary file 1 — Supplementary Material 1. [file 12872_2024_3834_MOESM1_ESM.zip › BNIP3-sham-DAPI.pdf]

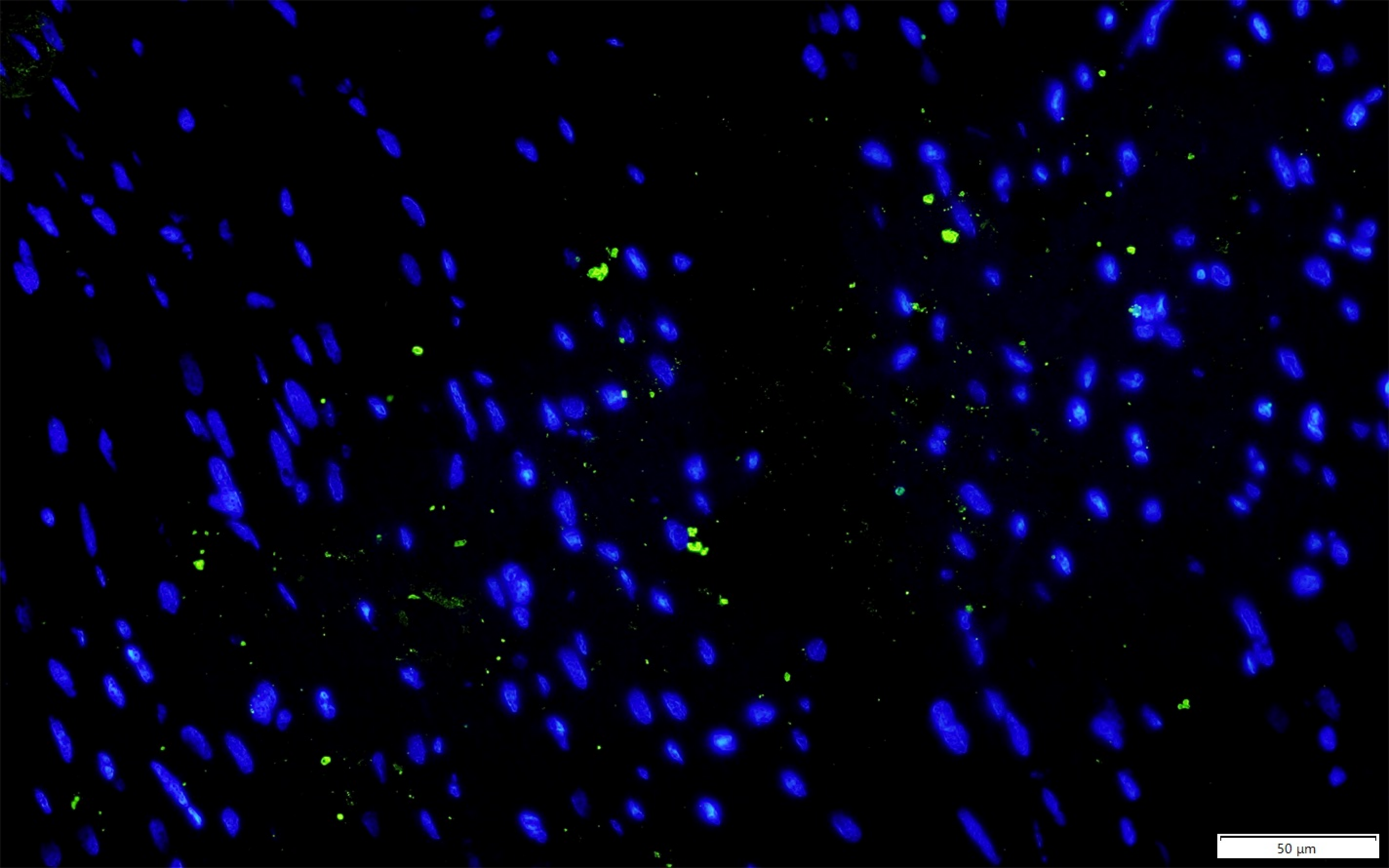

50  $\mu\text{m}$

Supplement: Supplementary file 1 — Supplementary Material 1. [file 12872_2024_3834_MOESM1_ESM.zip › BNIP3-sham-merge.pdf]

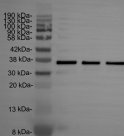

Supplement: Supplementary file 1 — Supplementary Material 1. [file 12872_2024_3834_MOESM1_ESM.zip › GAPDH-H9C2.pdf]

~200 kDa  
~140 kDa  
~110 kDa  
~90 kDa  
~68 kDa  
~55 kDa  
~40 kDa  
~30 kDa  
~20 kDa  
~13 kDa  
~8 kDa

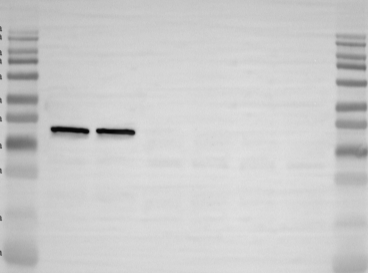

Supplement: Supplementary file 1 — Supplementary Material 1. [file 12872_2024_3834_MOESM1_ESM.zip › GAPDH-myocardial tissue.pdf]

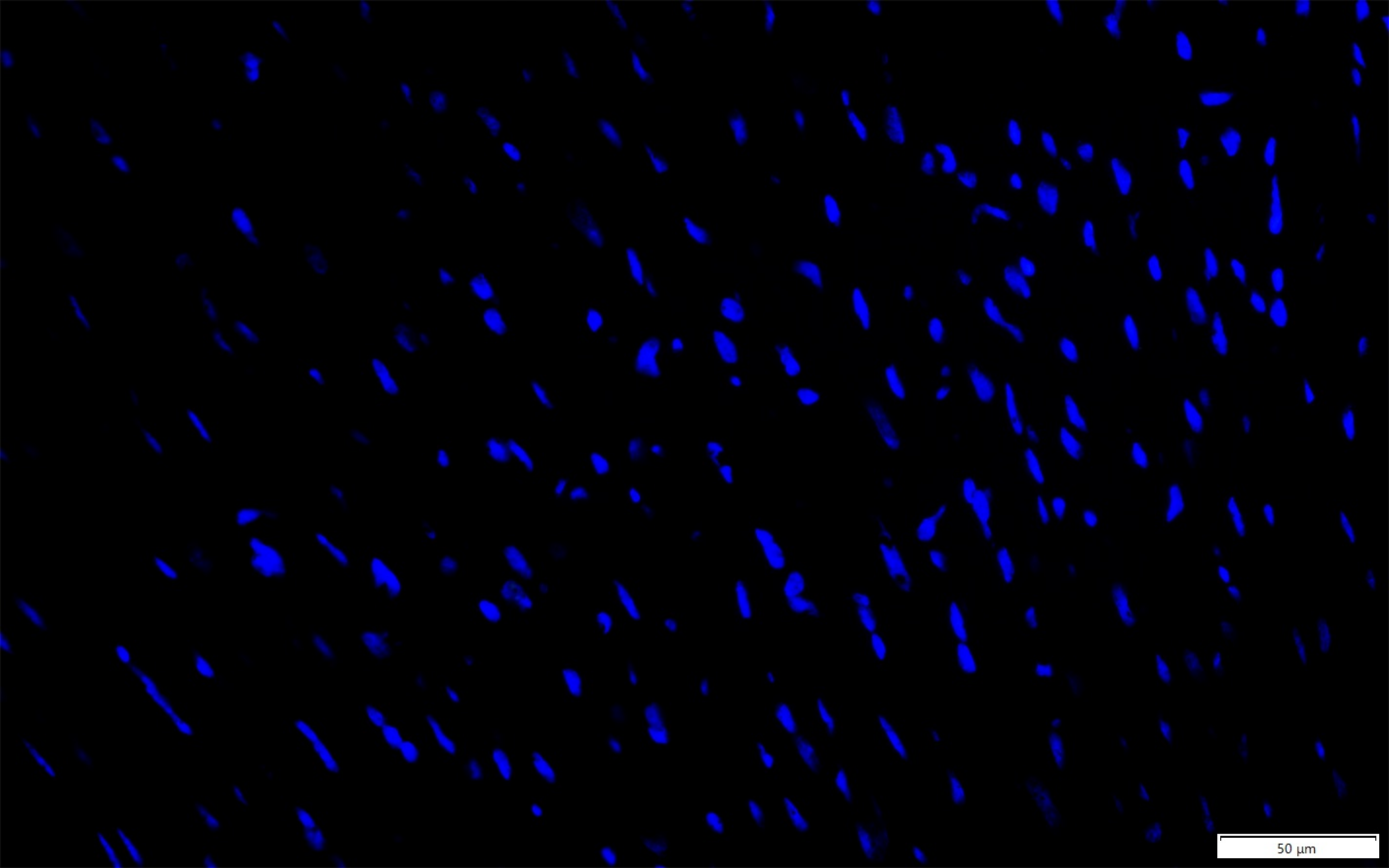

Supplement: Supplementary file 1 — Supplementary Material 1. [file 12872_2024_3834_MOESM1_ESM.zip › HIF1a-MIRI-DAPI.pdf]

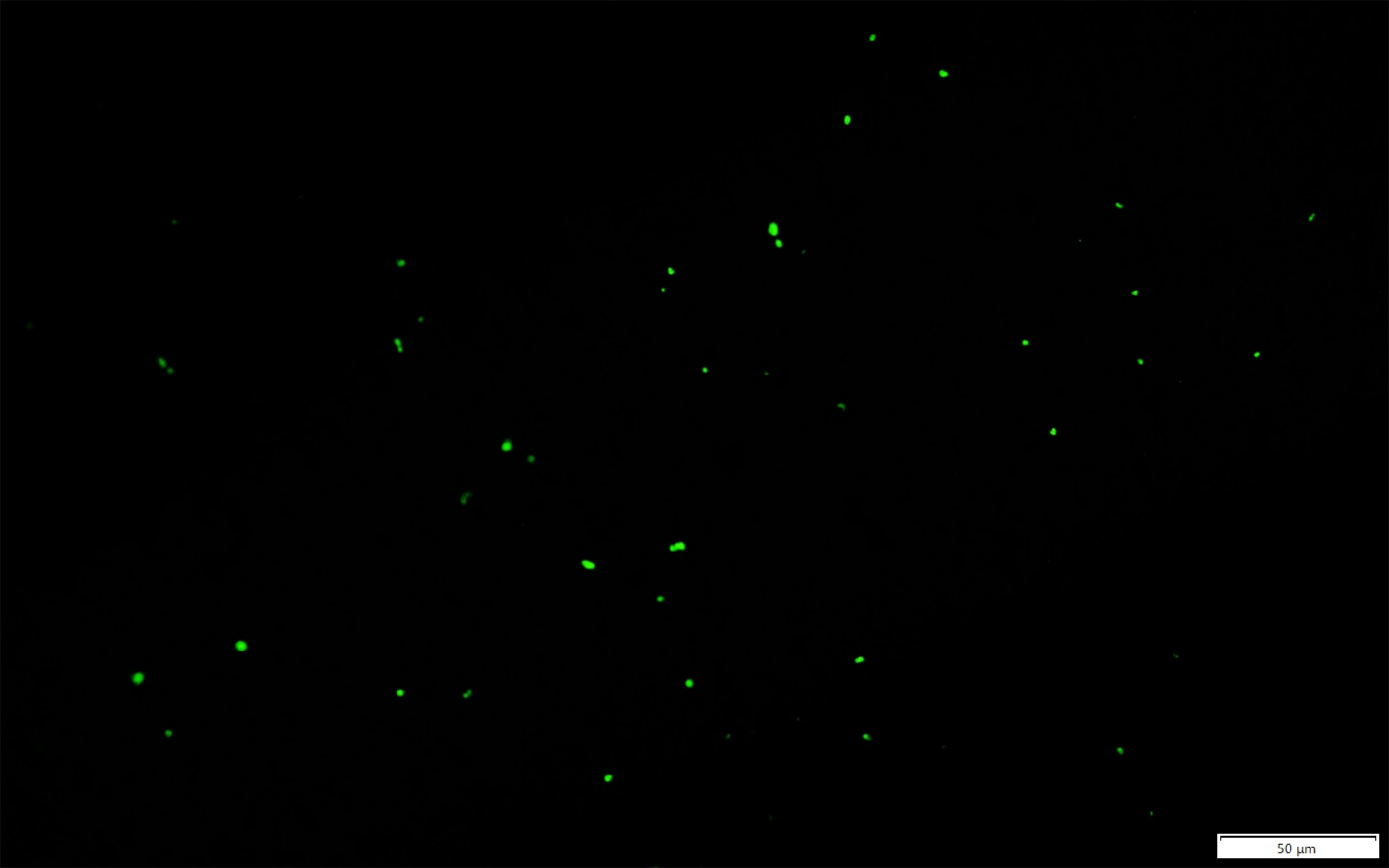

50  $\mu\text{m}$

Supplement: Supplementary file 1 — Supplementary Material 1. [file 12872_2024_3834_MOESM1_ESM.zip › HIF1a-MIRI-hif1a.pdf]

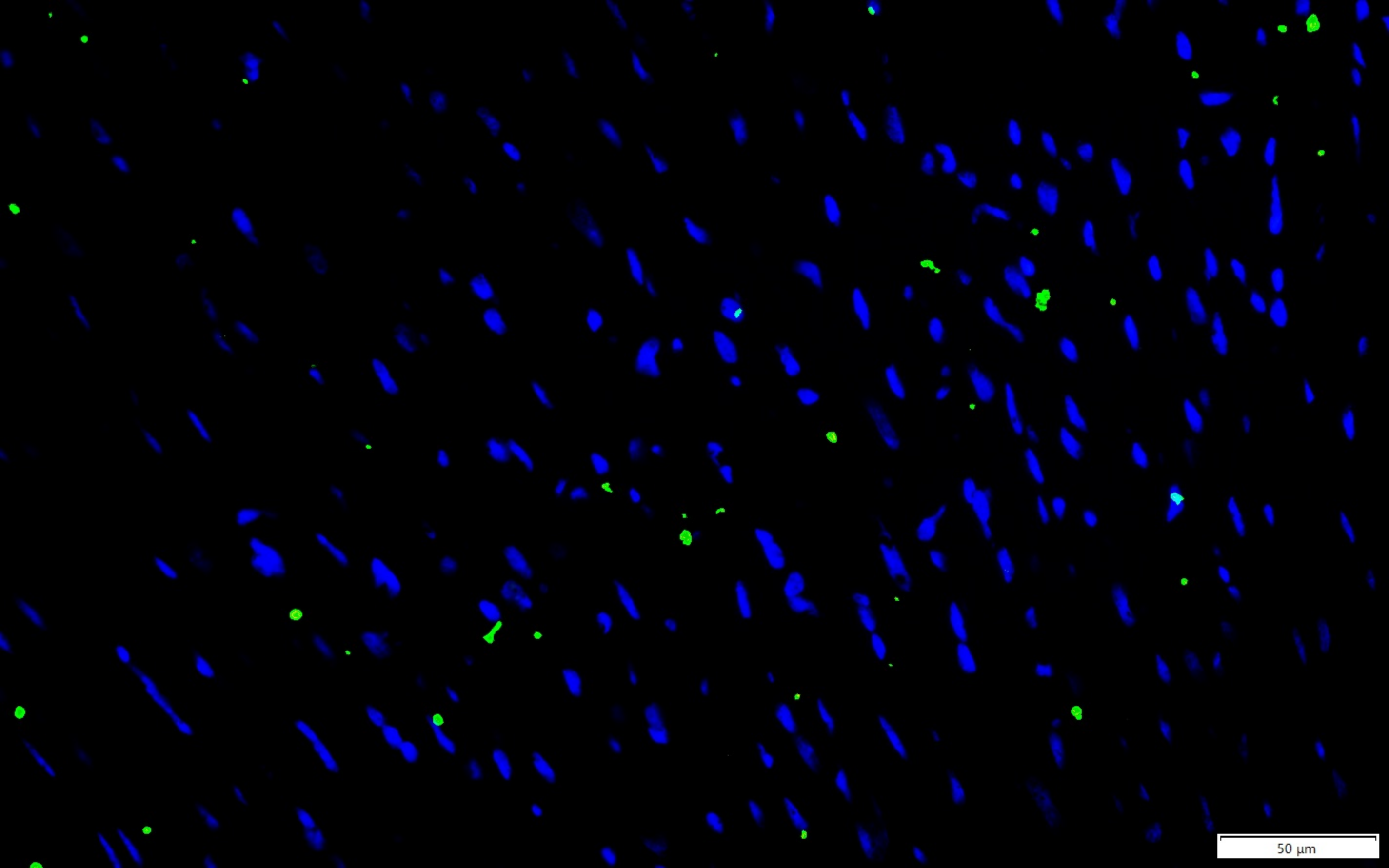

50 μm

Supplement: Supplementary file 1 — Supplementary Material 1. [file 12872_2024_3834_MOESM1_ESM.zip › HIF1a-MIRI-merge.pdf]

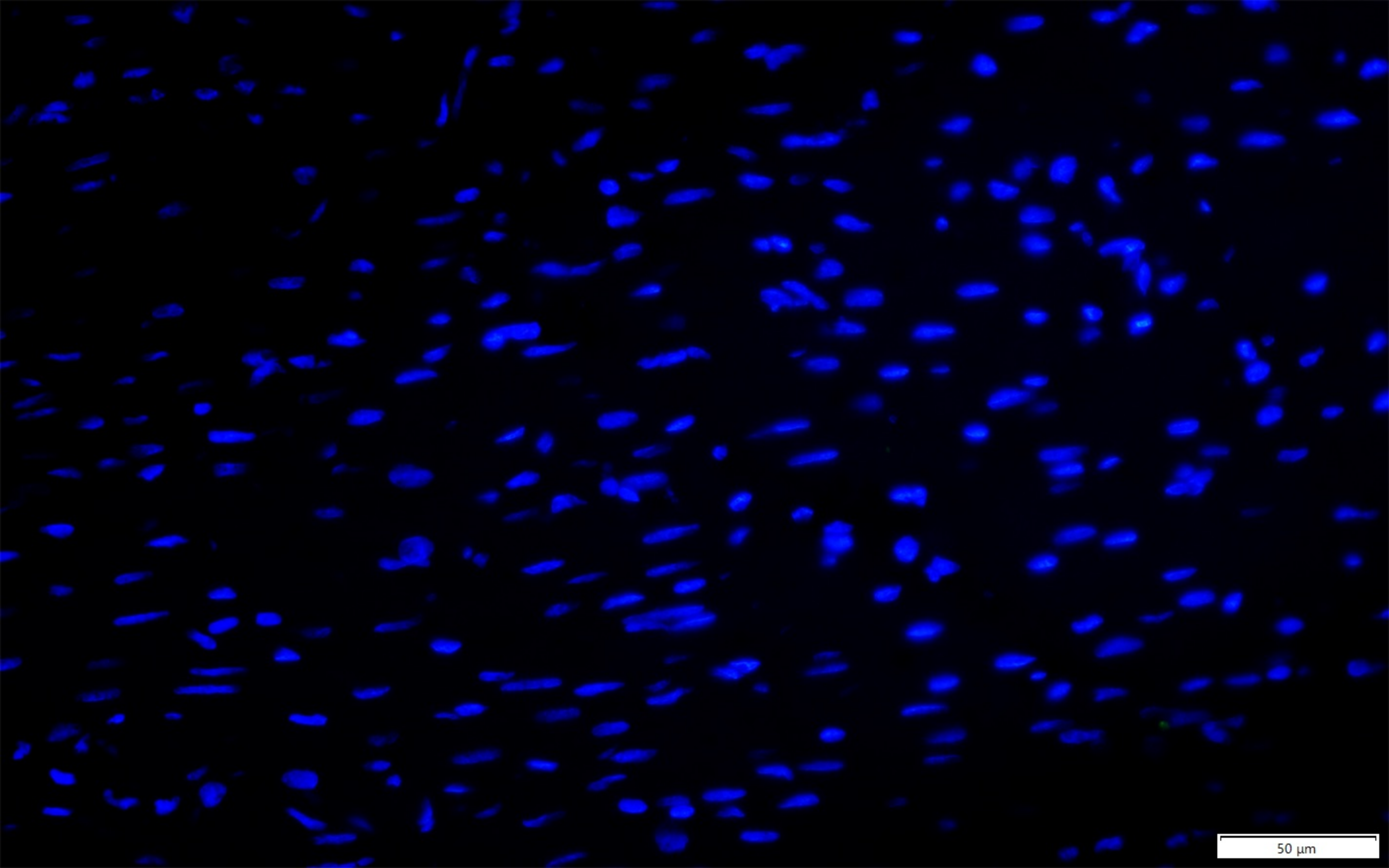

50 μm

Supplement: Supplementary file 1 — Supplementary Material 1. [file 12872_2024_3834_MOESM1_ESM.zip › HIF1a-sham-DAPI.pdf]

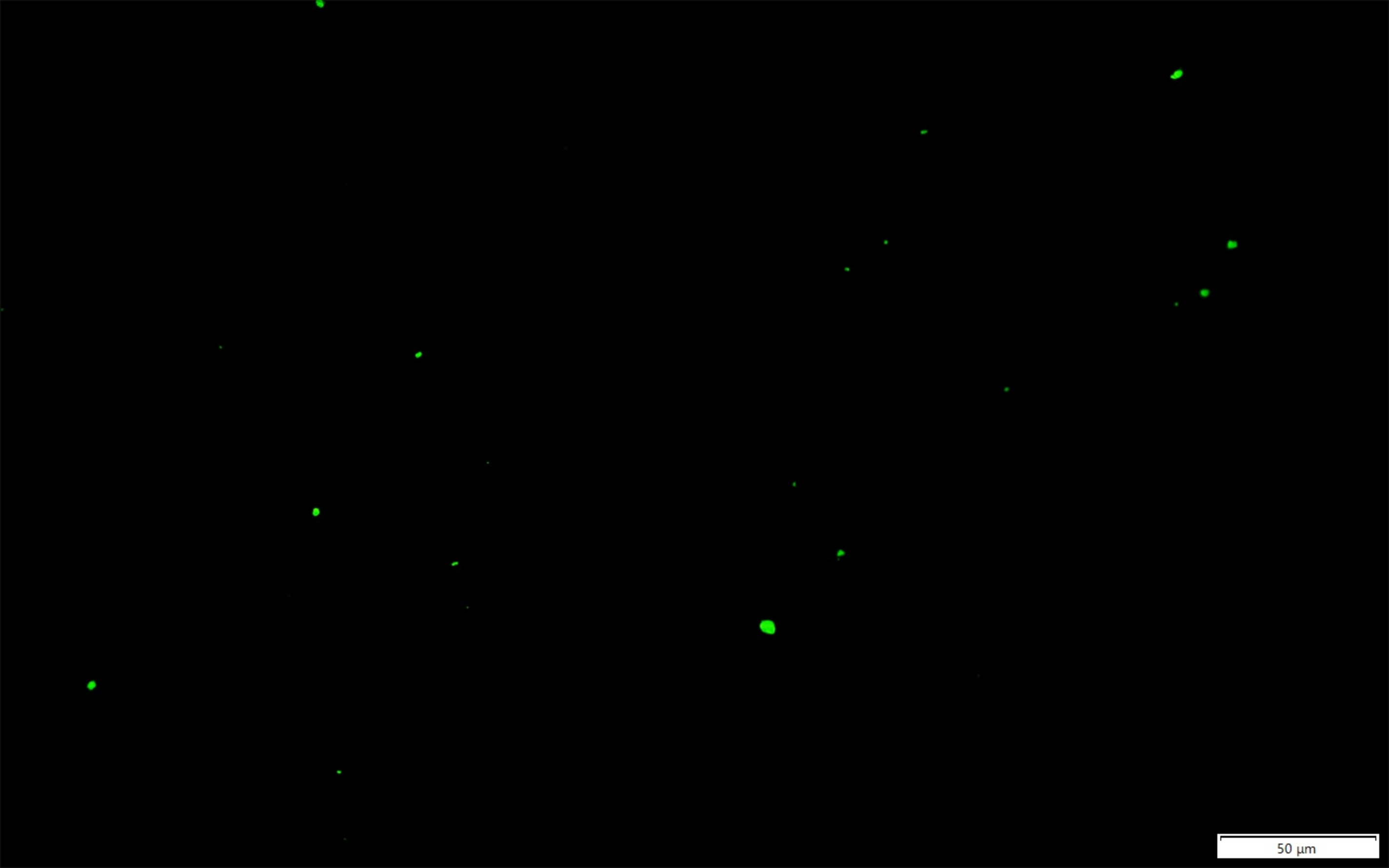

Supplement: Supplementary file 1 — Supplementary Material 1. [file 12872_2024_3834_MOESM1_ESM.zip › HIF1a-sham-hif1a.pdf]

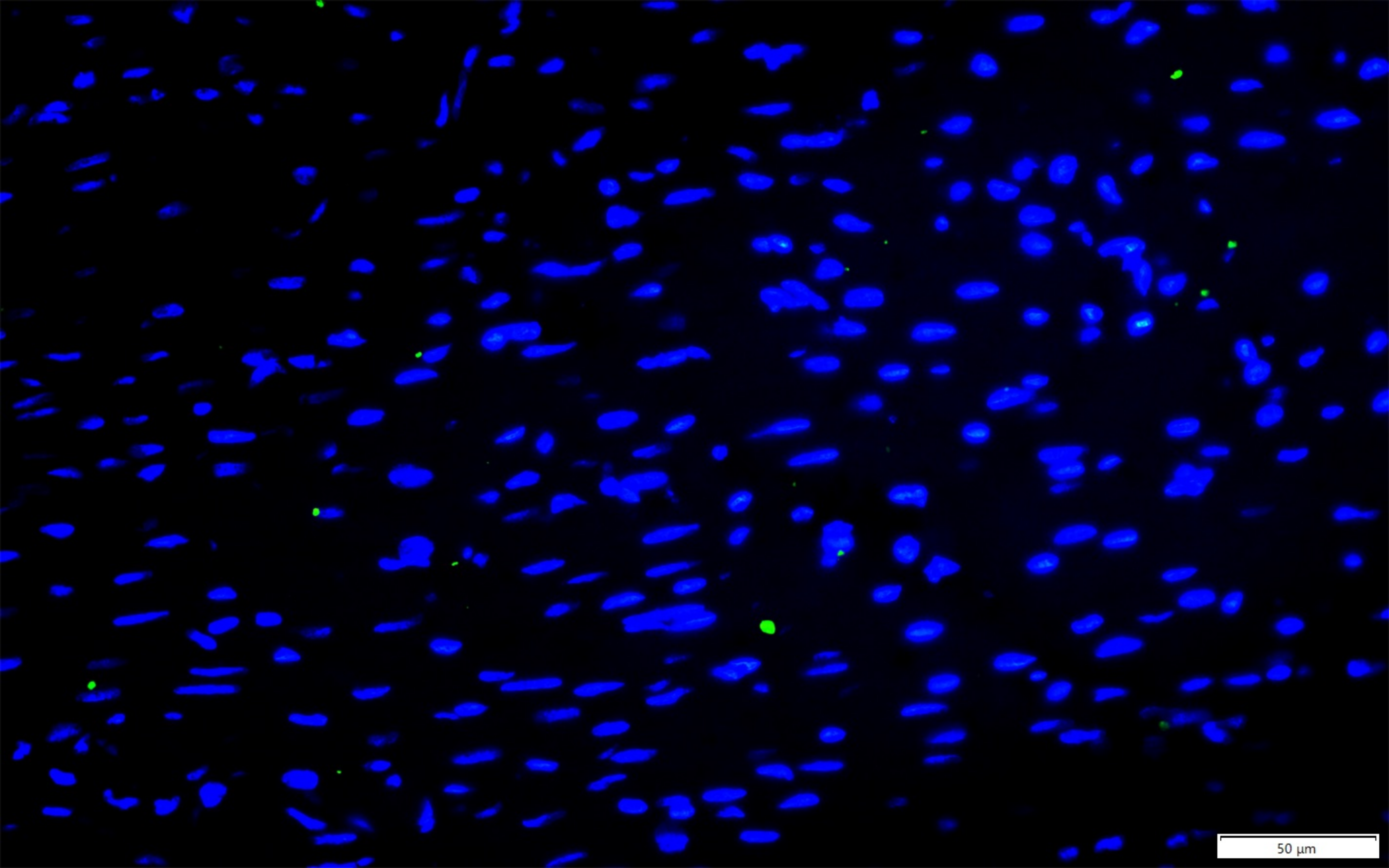

50  $\mu\text{m}$

Supplement: Supplementary file 1 — Supplementary Material 1. [file 12872_2024_3834_MOESM1_ESM.zip › HIF1a-sham-merge.pdf]

195 kDa-  
135 kDa-  
105 kDa-  
90 kDa-  
63 kDa-  
45 kDa-  
40 kDa-  
30 kDa-  
20 kDa-  
13 kDa-  
8 kDa-

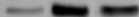

Supplement: Supplementary file 1 — Supplementary Material 1. [file 12872_2024_3834_MOESM1_ESM.zip › HIF1-H9C2.pdf]

190 kDa-  
130 kDa-  
100 kDa-  
90 kDa-  
58 kDa-  
42kDa-  
38 kDa-  
30 kDa-  
20 kDa-  
13 kDa-  
8 kDa-

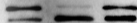

Supplement: Supplementary file 1 — Supplementary Material 1. [file 12872_2024_3834_MOESM1_ESM.zip › LC3-H9C2.pdf]

~200 kDa  
~140 kDa  
~110 kDa  
~90 kDa  
~68 kDa  
~55 kDa  
~40 kDa  
~30 kDa  
~20 kDa  
~13 kDa  
~8 kDa

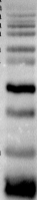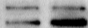

Supplement: Supplementary file 1 — Supplementary Material 1. [file 12872_2024_3834_MOESM1_ESM.zip › LC3-myocardial tissue.pdf]
